# Supplementary material for: Development of the TrAnsparent ReportinG of observational studies Emulating a Target trial (TARGET) guideline
Source: BMJ Open. 2023 Sep 12;13(9):e074626. doi: 10.1136/bmjopen-2023-074626 (PMC10503363; doi:10.1136/bmjopen-2023-074626)
Supplement: Supplementary data [file bmjopen-2023-074626supp001.pdf]

Supplementary Material

Supplementary Material 1: TARGET working group members (alphabetical)

Steering committee
Dr Aidan G. Cashin
Mr Harrison J. Hansford
Prof Miguel A. Hernán
Dr Hopin Lee
Dr Matthew D. Jones
Prof James H. McAuley
A/Prof Sonja A. Swanson

Project team
A/Prof Issa J. Dahabreh
A/Prof Barbra A. Dickerman
Prof Matthias Egger
Dr Xabier Garcia-Albeniz
Prof Robert M. Golub
A/Prof Nazrul Islam
A/Prof Sara Lodi
A/Prof Margarita Moreno-Betancur
Prof Sallie A. Pearson
Prof Sebastian Schneeweiss
Prof Jonathan A. C. Sterne
Dr Melissa K. Sharp
Prof Elizabeth A. Stuart

BMJ Publishing Group Limited (BMJ) disclaims all liability and responsibility arising from any reliance placed on this supplemental material which has been supplied by the author(s)

Supplementary Material 2: Complete search strategies for all databases

Medline

1 (emulat* adj5 trial?).mp.
2 (target adj (trial? or experiment?)).mp.
3. (observational adj (stud* or research or data)).mp.
4. ((real world or rwd) adj2 (stud* or research or data)).mp.
5. (routine* adj2 data).mp.
6. (comparative effectiveness adj2 (stud* or research or data)).mp.
7. (emulat* or propensity score? or (causal adj2 (inference? or analys?s or effect*))).mp.
8. 3 or 4 or 5 or 6 or 7
9. 2 and 8
10. (target adj (trial? or experiment?)).ti.
11. 1 or 9 or 10
Filtered for time (2012-2022) manually after search

Embase

1. (emulat* adj5 trial?).mp.
2. (target adj (trial? or experiment?)).mp.
3. (observational adj (stud* or research or data)).mp.
4. ((real world or rwd) adj2 (stud* or research or data)).mp.
5. (routine* adj2 data).mp.
6. (comparative effectiveness adj2 (stud* or research or data)).mp.
7. (emulat* or propensity score? or (causal adj2 (inference? or analys?s or effect*))).mp.
8. 3 or 4 or 5 or 6 or 7
9. 2 and 8
10. (target adj (trial? or experiment?)).ti.
11. 1 or 9 or 10

psycINFO

noft(target trial emulat*) OR ((noft(real world data) OR (noft(emulat* trial)) OR noft(observational) OR noft(routine* data)) AND noft(comparative effective*) AND noft(causal infer*))

Web of Science

(TI=(emulat* trial)) OR (TI=(real world data) OR TI=(routine* data) OR TI=(comparative effectiveness study comparative effectiveness research or comparative effectiveness data) OR (TI=(emulat* or propensity score?) AND TI=(causal inference or causal analysis or causal effect*))) AND ALL=(target trial or emulat* or target trial emulation)
