# Supplementary material for: Development of the TrAnsparent ReportinG of observational studies Emulating a Target trial (TARGET) guideline
Source: BMJ Open. 2023 Sep 12;13(9):e074626. doi: 10.1136/bmjopen-2023-074626 (PMC10503363; doi:10.1136/bmjopen-2023-074626)
Supplement: Author's manuscript [file bmjopen-2023-074626.draft_revisions.pdf]

BMJ Open is committed to open peer review. As part of this commitment we make the peer review history of every article we publish publicly available.

When an article is published we post the peer reviewers' comments and the authors' responses online. We also post the versions of the paper that were used during peer review. These are the versions that the peer review comments apply to.

The versions of the paper that follow are the versions that were submitted during the peer review process. They are not the versions of record or the final published versions. They should not be cited or distributed as the published version of this manuscript.

BMJ Open is an open access journal and the full, final, typeset and author-corrected version of record of the manuscript is available on our site with no access controls, subscription charges or pay-per-view fees (http://bmjopen.bmj.com).

If you have any questions on BMJ Open's open peer review process please email info.bmjopen@bmj.com

BMJ Open

Development of the Transparent Reporting of Observational Studies Emulating a Target Trial (TARGET) Guideline

Journal:	*BMJ Open*
Manuscript ID	bmjopen-2023-074626
Article Type:	Protocol
Date Submitted by the Author:	12-Apr-2023
Complete List of Authors:	Hansford, Harrison; University of New South Wales; Neuroscience Research Australia<br>Cashin, Aidan; Neuroscience Research Australia, Centre for Pain IMPACT; University of New South Wales, School of Health Sciences, Faculty of Medicine and Health<br>Jones, Matthew; University of New South Wales; Neuroscience Research Australia, Centre for Pain IMPACT<br>Swanson, Sonja; University of Pittsburgh, Department of Epidemiology; Harvard T.H. Chan School of Public Health, CAUSALab, Department of Epidemiology<br>Islam, Nazrul; University of Oxford, Big data institute; University of Southampton, Faculty of Medicine<br>Dahabreh, Isa; , Beth Israel Deaconess Medical Center and Harvard Medical School, Richard A. and Susan F. Smith Center for Outcomes Research; Harvard T.H. Chan School of Public Health, CAUSALab, Department of Epidemiology, Department of Biostatistics<br>Dickerman, Barbra; Harvard TH Chan School of Public Health, CAUSALab; Harvard TH Chan School of Public Health, Department of Epidemiology<br>Egger, Matthias; University of Bern, Institute of Social & Preventive Medicine; University of Cape Town Faculty of Health Sciences, Centre for Infectious Disease Epidemiology and Research<br>Garcia-De-Albeniz, Xavier ; Harvard University T H Chan School of Public Health, CAUSALab; RTI Health Solutions Barcelona<br>Golub, Robert ; Northwestern University Feinberg School of Medicine<br>Lodi, Sara; Harvard TH Chan School of Public Health, CAUSALab; Boston University School of Public Health, Department of Biostatistics<br>Moreno-Betancur , Margarita ; Murdoch Children's Research Institute, Melbourne, Clinical Epidemiology & Biostatistics Unit; The University of Melbourne, Department of Paediatrics<br>Pearson, Sallie-Anne; University of New South Wales, School of Population Health<br>Schneeweiss , Sebastian ; Harvard Medical School, Boston, USA, Division of Pharmacoepidemiology, Department of Medicine, Brigham & Women's Hospital,<br>Sterne, Jonathan; University of Bristol, Department of Population Health Sciences; NIHR Bristol Biomedical Research Centre<br>Sharp, Melissa; RCSI University of Medicine and Health Sciences, Department of General Practice<br>Stuart, Elizabeth A; Johns Hopkins Bloomberg School of Public Health, Department of Mental Health

Hernan, M; Harvard School of Public Health, CAUSALab; Harvard T.H Chan School of Public Health, Department of Epidemiology, Department of Biostatistics
Lee, Hopin; University of Exeter Medical School
McAuley, James; University of New South Wales, School of Health Sciences, Faculty of Medicine and Health; Neuroscience Research Australia, Centre for Pain IMPACT

Keywords: EPIDEMIOLOGY, Retrospective Studies, STATISTICS & RESEARCH METHODS

SCHOLARONE™
Manuscripts

Development of the Transparent Reporting of Observational Studies Emulating a Target Trial (TARGET) Guideline

Harrison J. Hansford[1,2], Aidan G. Cashin[1,2], Matthew D. Jones[1,2], Sonja A. Swanson[3,8,9], Nazrul Islam[5,6], Issa J. Dahabreh[7,8, 9,10], Barbra A. Dickerman[8,9], Matthias Egger[11,12,13], Xabier Garcia-Albeniz[8, 14], Robert M. Golub[15], Sara Lodi[8,16], Margarita Moreno-Betancur[17,18], Sallie-Anne Pearson[19], Sebastian Schneeweiss[20], Jonathan A. C. Sterne[21,22,23], Melissa K. Sharp[24], Elizabeth A. Stuart[25], Miguel A. Hernán[8,9,10], Hopin Lee[26], James H. McAuley[1,2]

1. School of Health Sciences, Faculty of Medicine and Health, University of New South Wales, Sydney, Australia
2. Centre for Pain IMPACT, Neuroscience Research Australia, Sydney, Australia
3. Department of Epidemiology, University of Pittsburgh, Pittsburgh, United States of America
5. Oxford Population Health, Big Data Institute, University of Oxford, Oxford, UK
6. Faculty of Medicine, University of Southampton, Southampton, UK
7. Richard A. and Susan F. Smith Center for Outcomes Research, Beth Israel Deaconess Medical Center and Harvard Medical School, Boston, MA, USA
8. CAUSALab, Harvard T.H. Chan School of Public Health, Boston, MA, USA
9. Department of Epidemiology, Harvard T.H. Chan School of Public Health, Boston, MA, USA
10. Department of Biostatistics, Harvard T.H. Chan School of Public Health, Boston, MA, USA
11. Institute of Social and Preventive Medicine, University of Bern, Bern, Switzerland
12. Centre for Infectious Disease Epidemiology and Research, Faculty of Health Sciences, University of Cape Town, Cape Town, South Africa
13. Population Health Sciences, Bristol Medical School, University of Bristol, Bristol, UK
14. RTI Health Solutions, Barcelona, Spain
15. Northwestern University Feinberg School of Medicine, Chicago, IL, USA
16. Department of Biostatistics, Boston University School of Public Health, Boston, MA, USA
17. Clinical Epidemiology & Biostatistics Unit, Murdoch Children's Research Institute, Royal Children's Hospital, 50 Flemington Rd, Parkville, Melbourne, VIC, Australia
18. Department of Paediatrics, The University of Melbourne, Parkville, Australia

For peer review only

For peer review only - http://bmjopen.bmj.com/site/about/guidelines.xhtml

19. School of Population Health, Faculty of Medicine and Health, UNSW Sydney, Sydney, Australia

20. Division of Pharmacoepidemiology, Department of Medicine, Brigham & Women's Hospital, Harvard Medical School, Boston, MA, USA

21. Department of Population Health Sciences, Bristol Medical School, University of Bristol, UK

22. NIHR Bristol Biomedical Research Centre, UK

23. Health Data Research UK South-West, Bristol, UK

24. Department of General Practice, RCSI University of Medicine and Health Sciences, Dublin, Ireland

25. Department of Mental Health, Johns Hopkins Bloomberg School of Public Health, Baltimore, MD, USA

26. University of Exeter Medical School, Exeter, UK

Corresponding Author

Harrison J Hansford

E: h.hansford@unsw.edu.au

School of Health Sciences, UNSW Sydney,

2052, Sydney, Australia

Words: 2809

For peer review only

Abstract

Background

Observational studies are increasingly being used to inform health decision-making when randomised trials are not feasible, ethical, or timely. The target trial approach provides a framework to help minimise common biases in observational studies that aim to estimate the causal effect of interventions. Incomplete reporting of studies using the target trial framework limits the ability for clinicians, researchers, patients, and other decision-makers to appraise, synthesise, and interpret findings to inform clinical and public health practice and policy. This paper describes the methods that we will use to develop the Transparent reporting of observational studies emulating a target trial (TARGET) reporting guideline.

Methods/design

The TARGET reporting guideline will be developed in five stages. The first stage will identify current target trial reporting practices by systematically reviewing published studies that explicitly emulated a target trial. The second stage will identify and refine items to be considered for inclusion in the TARGET guideline by consulting content experts using two online surveys. The third stage will prioritise and consolidate key items to be included in the TARGET guideline at a consensus meeting of TARGET investigators. The fourth stage will produce and pilot-test the TARGET guideline and

For peer review only

explanation and elaboration document. The fifth stage will disseminate the TARGET guideline and resources via journals, conferences, and courses.

Ethics and Dissemination

Ethical approval for the survey to be conducted has been attained (HC220536). The TARGET guideline will be disseminated widely and should improve the transparency and completeness of reporting in studies using the target trial framework.

Key words: target trial emulation, causal inference, reporting guideline, observational studies

Strengths and Limitations

- The TARGET reporting guideline will be developed according to recommendations for health research reporting guidelines
- The TARGET working group has been established to include stakeholders from a variety of backgrounds

For peer review only

Introduction

Observational studies can provide evidence on the causal effects of interventions when it is not feasible, ethical, or timely to conduct a relevant randomised trial. However, making causal inferences from observational data is challenging due to confounding and design-related biases such as selection bias and immortal time bias. [1 2] Design-related biases can be avoided using the target trial framework. [3 4] The framework involves the specification of the hypothetical randomised pragmatic trial — the target trial — that would ideally be conducted and how this trial might be emulated using observational data. [3 4] The two stages of the target trial framework are 1) specification of the target trial, and 2) emulation of the target trial. [3 4] Using observational data to mimic a randomised experiment was proposed in the mid 20th century, [5-8] and extended to time-varying treatments by Robins in 1986. [9]

The value of using the target trial framework to design the analysis of observational studies has been recognised by international regulatory bodies in the field of medicine and health, [10-14] and the framework underpins the widely-used ROBINS-I tool for assessing risk of bias in non-randomised studies of interventions. [15] Studies that are explicit in using the target trial framework have been published with increasing frequency in leading general medical and specialty journals. [16-21]

Application of the target trial framework requires the complete specification of the target trial protocol and its emulation (Figure 1). [3] Hernán & Robins [3] provide a

For peer review only

template for specifying a target trial and its emulation; however, there is currently no detailed guidance on reporting a study designed to emulate a target trial. Incomplete reporting of these studies limits the ability of clinicians, researchers, patients, and other decision-makers to appraise and synthesise findings or interpret them to inform clinical and public health practice and policy. A reporting guideline that expands upon the initial target trial emulation template [3] is needed to provide authors with comprehensive recommendations on how to completely and transparently report a study emulating a target trial.

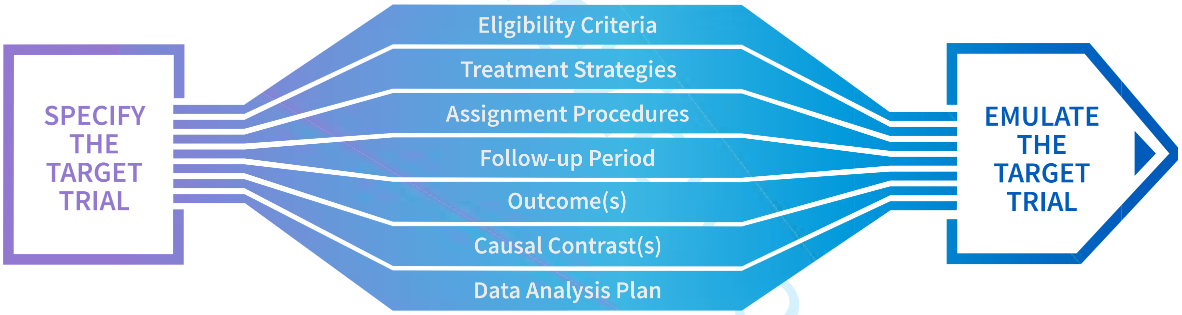

Figure 1. Elements relevant to both the specification and emulation of the target trial described by Hernán & Robins (3)

To address this gap, we outline the processes and methods that used to develop a reporting guideline for studies emulating a target trial – TARGET (Transparent reporting of observational studies emulating a target trial).

For peer review only

For peer review only - http://bmjopen.bmj.com/site/about/guidelines.xhtml

Objective

The objective of the TARGET guideline is to provide guidance on the minimum set of items that should be reported to provide a clear and transparent account of observational studies that investigate the comparative effectiveness and safety of health interventions explicitly using the target trial framework.

Methods/design

We will develop the TARGET Guideline in five stages following recommendations for the development of health research reporting guidelines (Figure 2). [22]

For peer review only

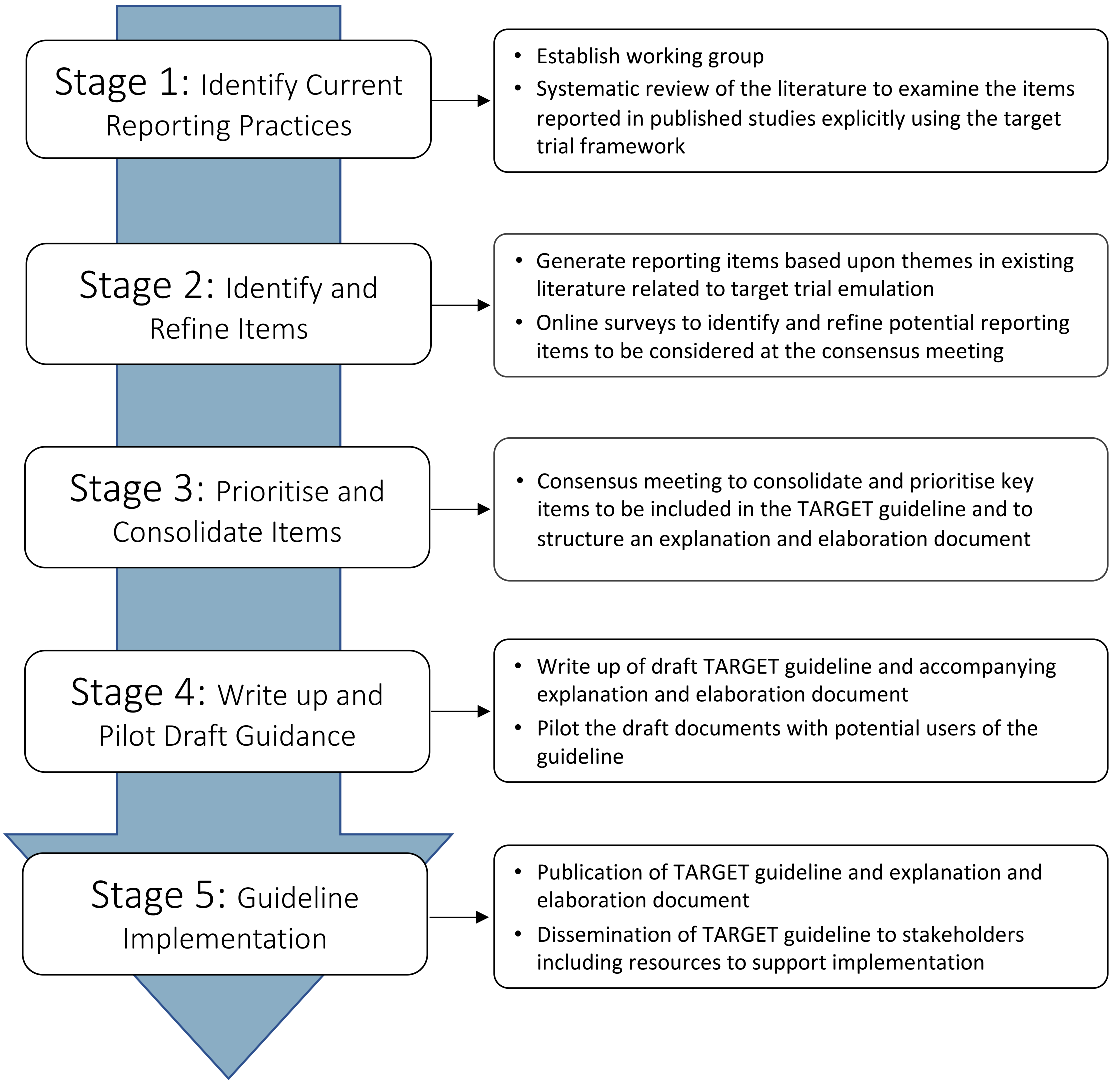

Figure 2. Workflow for the development of the TARGET guideline

only

TARGET working group

The TARGET working group is made up of the steering committee and project team (Supplementary Material 1). The group was established to collate expertise on target trial emulation methodology, epidemiology, clinical trials, biostatistics, reporting guideline development, and knowledge of regulatory and journal editorial processes. The working group will oversee recruitment of participants for Stages 2 and 3 and contribute to writing and disseminating the guideline documents.

For peer review only - http://bmjopen.bmj.com/site/about/guidelines.xhtml

Stage 1: Identify current reporting practices

The systematic review aims to assess whether and how important items are reported by published studies explicitly emulating a target trial and whether reporting guidance (e.g., STROBE [23]) was used. The protocol for this systematic review was registered on the Open Science Framework on 13 March 2022 (osf.io/uj56m).

Databases, eligibility, and search terms

We will search Medline, EMBASE, PsycINFO and Science Citation Index for observational studies that stated in their methods that they explicitly emulated a target trial. We will exclude studies not written in English, not in the field of medicine and health, not conducted in humans, or not observational designs. Many observational studies may implicitly use the framework of a randomised trial. However, to be included in this review studies must be explicit in their attempt to emulate a target trial (e.g., stated 'target trial emulation' in the article). To identify eligible studies, we developed a literature search in collaboration with an expert librarian at the University of Oxford. Our approach used sensitive search terms including emulat*, target trial, observational data, real-world data, comparative effectiveness, and causal inference, to try to capture all papers explicitly emulating a target trial. The complete search strategy is in Supplementary Material 2. In duplicate, independent reviewers will conduct title, abstract, and full text screening. We will resolve disagreements between reviewers through discussion.

For peer review only

For peer review only - http://bmjopen.bmj.com/site/about/guidelines.xhtml

Data Extraction

We will extract items regarded by the steering committee as potentially important for the reporting of a target trial emulation, including those outlined by Hernán and Robins, 2016. [3] Two independent reviewers will extract information on study authors, year of publication, journal, sub-field of medicine, study design, sample size, intervention, comparison group, outcomes assessed, and whether the study was prospectively registered. We will extract items relevant to the methods and results of the target trial emulation, including whether and how all components of the protocol of the proposed target trial, and how they were emulated, were specified (i.e., eligibility criteria, treatment strategies, assignment procedures, follow-up period, outcome(s), causal contrast(s), and data analysis plan). We will enter data into a standardised data extraction form which two authors will pilot with a selection of included studies. We will resolve disagreements in data extraction between reviewers through discussion, or where necessary, consultation with a third reviewer.

Data analysis

We will use R [24] for all data analyses. Categorical variables will be summarised using frequencies and percentages. Continuous variables will be summarised using mean and standard deviation, or median and interquartile range, as appropriate.

Outcomes of the systematic review

For peer review only

For peer review only - http://bmjopen.bmj.com/site/about/guidelines.xhtml

The systematic review will provide evidence on reporting in studies explicitly emulating a target trial. The findings will inform the online surveys (Stage 2) and the consensus meeting (Stage 3). We will submit the findings of this review for publication and all data and code made publicly available.

Stage 2: Identify and refine items for the TARGET guideline

We will conduct two online surveys to generate a list of candidate items that add detail to each of the protocol elements in Figure 1.

Ethics

Ethical approval has been obtained for the online surveys from the University of New South Wales Human Research Ethics Committee (HC220536).

Selection of initial items

The steering group will develop a list of key items, informed by the systematic review (Stage 1), and the target trial framework described by Hernán & Robins (3), thought important for the conduct and reporting target trial emulations (Figure 1). Other potential sources of items include: published guidance for observational studies and randomised controlled trials, the ROBINS-I tool, [15] and studies that describe items that may be important for the conduct or reporting of target trial emulations.

Participants

For peer review only - http://bmjopen.bmj.com/site/about/guidelines.xhtml

Members of the TARGET working group (Supplementary Material 1) will be invited to participate in the surveys.

Procedure

We will host two online surveys using REDCap. [25 26] We will send each online survey via email to the participants. We will ask participants to rate the importance of each potential reporting item on a 9-point Likert scale (1, "not important", to 9, "critically important"). Participants will have the opportunity to provide suggestions or modifications to the wording of items as well as suggest additional items or make other comments.

In the second survey, we will send participants a summary of the results for each potential reporting item (mean scores and standard deviations, median scores and interquartile ranges, and histograms), their own score for each item, and any comments from participants on each item from the first survey. We will also present any new items and suggested modifications to items. We will then invite participants to re-score the importance of each item, and score any additional items, considering the aggregated ratings. Participants will have the opportunity to provide additional feedback on each item in the form of open ended responses.

Analysis

For peer review only

Continuous variables will be summarised using mean and standard deviation, and median and interquartile range. We will analyse the free-text responses from the first and second surveys using an inductive approach, in which we will use reflexive thematic analysis to identify, organise and generate codes, and then identify themes found within the dataset. These data will contribute to the creation of new items and modification of existing items to be included in the subsequent survey.

Outcome of the online surveys

We will generate a preliminary list of items with corresponding ratings of importance to be considered in the TARGET guideline at the consensus meeting (Stage 3). We will also generate qualitative insights to guide item refinement and prioritisation in preparation for the consensus meeting.

Stage 3 – Consolidate and prioritise key items to be included in the TARGET guideline

A consensus meeting will finalise reporting items for the TARGET guideline. [22] The consensus meeting will follow suggested methods for developing reporting guidelines [22], including guidance for consensus-based methods currently being developed which we will use if they become available. [27]

Process

We will invite stakeholders identified by the working group to participate in a two-day consensus meeting. The TARGET working group will ensure that the expertise of

For peer review only - http://bmjopen.bmj.com/site/about/guidelines.xhtml

consensus meeting participants includes target trial emulation methodology, epidemiology, clinical trials, biostatistics, reporting guideline development, and regulatory and journal editorial processes. Prior to the consensus meeting, the core team will provide attendees with evidence from the systematic review (Stage 1) and findings from the online surveys (Stage 2) including a draft of the items proposed for inclusion in the guideline. We will present the findings from Stage 1 and 2 at the consensus meeting. A member of the TARGET working group will facilitate a structured discussion on the rationale for including items from the online surveys. If there are disagreements, they will first be debated and, if disagreements remain, we will hold an anonymised vote to establish the importance of including the item in the guideline. For the anonymised vote, a simple majority will be sufficient to guide the inclusion/exclusion of an item. The meeting will conclude with discussion about the content and production of relevant documents (TARGET guideline, draft explanation and elaboration document) as well as strategies for dissemination and implementation. Following the conclusion of the consensus meeting, we will circulate a report on the outcome to the meeting participants for review and approval.

Stage 4 – Development and piloting of the draft TARGET guideline and explanation and elaboration document

Stage 4 involves drafting the TARGET guideline and accompanying explanation and elaboration document to ensure that the wording and content of the documents are clear, precise, and suitable for all identified stakeholders. The purpose of the

For peer review only

explanation and elaboration document is to explain each item by providing background information, a rationale, and clear reporting examples from published target trial emulations. We will design the explanation and elaboration document to facilitate adherence to the TARGET guideline by clarifying the importance of each item, highlighting relevant reporting issues and providing examples to assist authors using the guideline. The consensus meeting participants may be asked to review and comment on the draft TARGET guideline and explanation and elaboration document.

We will evaluate the TARGET guideline by piloting the proposed guideline and the explanation and elaboration document with 20-30 expert methodologists and potential users of TARGET, identified from TARGET working group networks. We will ask participants to provide general feedback on accessibility and usability, and to identify possible reporting items that might have been overlooked. We will also ask for specific feedback about the utility and clarity of each TARGET item. We will collect data through online surveys, hosted by REDCap. [25 26] We will incorporate feedback from the piloting exercise into the final guideline and explanation and elaboration document, as required. If suggested revisions are extensive, we will conduct a further round of piloting.

For peer review only

Stage 5 – Guideline implementation

The goal of the final stage of guideline development is to maximise reach and use of the TARGET guideline. The TARGET working group will guide the dissemination

strategy with advice from consensus meeting participants. We aim to publish the TARGET guideline and the explanation and elaboration document and disseminate the findings through traditional and social media. We will engage journal editors and funding agencies to encourage TARGET guideline endorsement alongside other published reporting guidance. We will publicly host the TARGET guideline and explanation and elaboration paper, and any other relevant material on a TARGET website. We will index the guideline on the Enhancing the QUAlity and Transparency Of health Research (EQUATOR) Network website. [28 29] We will create online resources including infographics, blog posts and podcasts, which will be available on the TARGET website. We will share the TARGET guideline with authors in the field, and at relevant scientific conferences and methodological courses.

Discussion

Studies that explicitly aim to emulate a target trial are increasingly published in the medical literature and are used to inform practice and policy decisions. A reporting guideline for these studies will facilitate comprehensive and transparent reporting and support accurate appraisal and implementation of study findings by researchers, clinicians, patients, and other decision-makers.

The TARGET guideline and supporting guidance material aim to improve the completeness and transparency of reporting of observational studies that aim to explicitly emulate a target trial in medical and health research. Although the focus is

For peer review only

on studies that explicitly use the target trial emulation framework much of the guidance will be applicable to studies using non-experimental comparison group designs to estimate causal effects. We will develop the TARGET guideline following accepted recommendations for the development of health research reporting guidelines to maximise the guidelines usefulness and usage. [22] We plan to use a structured dissemination approach to maximise uptake of the TARGET guideline and will ensure that the guideline is freely and easily accessible.

For peer review only

Declarations

Ethics approval and consent to participate

Not Applicable

Consent for publication

All authors consent to publication of this manuscript

Availability of data and materials

Not applicable

Funding

There was no specific funding for this study. HJH was supported by an Australian National Health and Medical Research Council (NHMRC) Postgraduate Scholarship, a PhD Top-Up Scholarship from Neuroscience Research Australia, and was a Neuroscience Research Australia PhD Pearl sponsored by Paul Salteri AO. AGC was supported by an Australian NHMRC Investigator Grant (ID 2010088). MM-B was supported by an Australian NHMRC Investigator Grant (ID 2009572). JMH was supported by an Australian NHMRC Investigator Grant (ID 2010128). BAD was supported by a grant from the National Institutes of Health (R00 CA248335). ME was supported by grants from the National Institutes of Health (R01 AI152772-01, 5U01-AI069924-05) and the Swiss National Science Foundation (32FP30-174281). NI was

For peer review only

For peer review only - http://bmjopen.bmj.com/site/about/guidelines.xhtml

supported by grants from the National Institute for Health and Care Research (HDRUK2022.0313) and the UK Office for National Statistics (2002563). MAH was supported by NIH grant R37 AI102634.

Competing interests

All authors declare no competing interests.

Author Contributions

HJH, AGC, MDJ, HL, JHM, conceived the idea for the project protocol. All authors contributed to the design and methodology of the project protocol. HJH and AGC wrote the first draft of the manuscript. All authors provided feedback, revised the manuscript and have read and approved the final version.

Acknowledgements

We acknowledge Nia Roberts, outreach librarian and information specialist at the University of Oxford for assistance designing the literature search.

Article Summary

Strengths and Limitations

For peer review only

376 **Abbreviations**

377

378 **EQUATOR:** Enhancing the QUAlity and Transparency Of health Research

379 **REDCap:** Research Electronic Data Capture

380 **STROBE:** Strengthening the Reporting of Observational Studies in Epidemiology

381 **TARGET:** TrAnsparent ReportinG of studies Emulating a Target trial

382

383

For peer review only

References

1. Hernán MA, Sauer BC, Hernández-Díaz S, et al. Specifying a target trial prevents immortal time bias and other self-inflicted injuries in observational analyses. *J Clin Epidemiol* 2016;79:70-75. doi: 10.1016/j.jclinepi.2016.04.014 [published Online First: 2016/05/31]
2. Dickerman BA, García-Albéniz X, Logan RW, et al. Avoidable flaws in observational analyses: an application to statins and cancer. *Nature Medicine* 2019;25(10):1601-06. doi: 10.1038/s41591-019-0597-x
3. Hernán MA, Robins JM. Using big data to emulate a target trial when a randomized trial is not available. *American journal of epidemiology* 2016;183(8):758-64.
4. Hernán MA. Methods of public health research—strengthening causal inference from observational data. *New England Journal of Medicine* 2021;385(15):1345-48.
5. Cochran WG. Observational studies. *Statistical papers in honor of George W Snedecor* 1972:77-90.
6. Dorn HF. Philosophy of inferences from retrospective studies. *American Journal of Public Health and the Nations Health* 1953;43(6_Pt_1):677-83.
7. Rubin DB. Estimating causal effects of treatments in randomized and nonrandomized studies. *Journal of educational Psychology* 1974;66(5):688.
8. Wold H. Causality and econometrics. *Econometrica: Journal of the Econometric Society* 1954:162-77.
9. Robins J. A new approach to causal inference in mortality studies with a sustained exposure period—application to control of the healthy worker survivor effect. *Mathematical modelling* 1986;7(9-12):1393-512.
10. Concato J, Stein P, Dal Pan GJ, et al. Randomized, observational, interventional, and real-world—What's in a name? *Pharmacoepidemiology and drug safety* 2020;29(11):1514-17.
11. Agency EM. European medicines agencies network strategy to 2025. The Netherlands: Health of Medicines Agencies, 2020.
12. National Institute for Health and Care Excellence. The NICE strategy 2021 to 2026, 2021.
13. Health Canada. Optimizing the Use of Real World Evidence to Inform Regulatory Decision-Making: Government of Canada, 2019.
14. Therapeutic Goods Administration. Real world evidence and patient reported outcomes in the regulatory context. https://www.tga.gov.au/review-real-world-evidence-and-patient-reported-outcomes: Australian Government, Department of Health, 2021.
15. Sterne JA, Hernán MA, Reeves BC, et al. ROBINS-I: a tool for assessing risk of bias in non-randomised studies of interventions. *BMJ* 2016;355:i4919. doi: 10.1136/bmj.i4919
16. Garcia-Albeniz XH, J.: Bretthauer, M.: Hernan, M. A. Effectiveness of screening colonoscopy to prevent colorectal cancer among medicare beneficiaries aged 70 to 79 years: A prospective observational study. *Annals of Internal Medicine* 2017 doi: http://dx.doi.org/10.7326/M16-0758
17. Mahévas M, Tran V-T, Roumier M, et al. Clinical efficacy of hydroxychloroquine in patients with covid-19 pneumonia who require oxygen: observational comparative study using routine care data. *BMJ* 2020;369:m1844. doi: 10.1136/bmj.m1844
18. Kaura A, Sterne JAC, Trickey A, et al. Invasive versus non-invasive management of older patients with non-ST elevation myocardial infarction (SENIOR-NSTEMI): a cohort

For peer review only

study based on routine clinical data. *The Lancet* 2020;396(10251):623-34. doi: 10.1016/S0140-6736(20)30930-2
19. Emilsson L, García-Albéniz X, Logan RW, et al. Examining Bias in Studies of Statin Treatment and Survival in Patients With Cancer. *JAMA Oncol* 2018;4(1):63-70. doi: 10.1001/jamaoncol.2017.2752
20. Chan You S, Krumholz HM, Suchard MA, et al. Comprehensive Comparative Effectiveness and Safety of First-Line β-Blocker Monotherapy in Hypertensive Patients: A Large-Scale Multicenter Observational Study. *Hypertension* 2021;77(5):1528-38. doi: 10.1161/hypertensionaha.120.16402 [published Online First: 20210329]
21. Caniglia EC, Robins JM, Cain LE, et al. Emulating a trial of joint dynamic strategies: An application to monitoring and treatment of HIV-positive individuals. *Stat Med* 2019;38(13):2428-46. doi: 10.1002/sim.8120 [published Online First: 20190318]
22. Moher D, Schulz KF, Simera I, et al. Guidance for developers of health research reporting guidelines. *PLoS medicine* 2010;7(2):e1000217.
23. Von Elm E, Altman DG, Egger M, et al. The Strengthening the Reporting of Observational Studies in Epidemiology (STROBE) statement: guidelines for reporting observational studies. *Bulletin of the World Health Organization* 2007;85:867-72.
24. R Core Team. R: A language and environment for statistical computing. 2013
25. Harris PA, Taylor R, Minor BL, et al. The REDCap consortium: Building an international community of software platform partners. *Journal of Biomedical Informatics* 2019;95:103208. doi: https://doi.org/10.1016/j.jbi.2019.103208
26. Harris PA, Taylor R, Thielke R, et al. Research electronic data capture (REDCap)—A metadata-driven methodology and workflow process for providing translational research informatics support. *Journal of Biomedical Informatics* 2009;42(2):377-81. doi: https://doi.org/10.1016/j.jbi.2008.08.010
27. Gattrell WT, Hungin AP, Price A, et al. ACCORD guideline for reporting consensus-based methods in biomedical research and clinical practice: a study protocol. *Research Integrity and Peer Review* 2022;7(1):3. doi: 10.1186/s41073-022-00122-0
28. The EQUATOR Network. EQUATOR Network - Enhancing the QUAlity and Transparency Of health Research 2022 [Available from: https://www.equator-network.org/2022.
29. Simera I, Moher D, Hoey J, et al. A catalogue of reporting guidelines for health research. *European journal of clinical investigation* 2010;40(1):35-53.

For peer review only

Supplementary Material

Supplementary Material 1: TARGET working group members (alphabetical)

Steering committee

Dr Aidan G. Cashin
Mr Harrison J. Hansford
Prof Miguel A. Hernán
Dr Hopin Lee
Dr Matthew D. Jones
Prof James H. McAuley
A/Prof Sonja A. Swanson

Project team

A/Prof Issa J. Dahabreh
A/Prof Barbra A. Dickerman
Prof Matthias Egger
Dr Xabier Garcia-Albeniz
Prof Robert M. Golub
A/Prof Nazrul Islam
A/Prof Sara Lodi
A/Prof Margarita Moreno-Betancur
Prof Sallie A. Pearson
Prof Sebastian Schneeweiss
Prof Jonathan A. C. Sterne
Dr Melissa K. Sharp
Prof Elizabeth A. Stuart

For peer review only

Supplementary Material 2: Complete search strategies for all databases

Medline

1 (emulat* adj5 trial?).mp.

2 (target adj (trial? or experiment?)).mp.

3. (observational adj (stud* or research or data)).mp.

4. ((real world or rwd) adj2 (stud* or research or data)).mp.

5. (routine* adj2 data).mp.

6. (comparative effectiveness adj2 (stud* or research or data)).mp.

7. (emulat* or propensity score? or (causal adj2 (inference? or analys?s or effect*))).mp.

8. 3 or 4 or 5 or 6 or 7

9. 2 and 8

10. (target adj (trial? or experiment?)).ti.

11. 1 or 9 or 10

Filtered for time (2012-2022) manually after search

Embase

1. (emulat* adj5 trial?).mp.

2. (target adj (trial? or experiment?)).mp.

3. (observational adj (stud* or research or data)).mp.

4. ((real world or rwd) adj2 (stud* or research or data)).mp.

5. (routine* adj2 data).mp.

6. (comparative effectiveness adj2 (stud* or research or data)).mp.

7. (emulat* or propensity score? or (causal adj2 (inference? or analys?s or effect*))).mp.

8. 3 or 4 or 5 or 6 or 7

9. 2 and 8

For peer review only

10. (target adj (trial? or experiment?)).ti.

11. 1 or 9 or 10

psycINFO

noft(target trial emulat*) OR ((noft(real world data) OR (noft(emulat* trial)) OR noft(observational) OR noft(routine* data)) AND noft(comparative effective*) AND noft(causal infer*))

Web of Science

(TI=(emulat* trial)) OR (TI=(real world data) OR TI=(routine* data) OR TI=(comparative effectiveness study comparative effectiveness research or comparative effectiveness data) OR (TI=(emulat* or propensity score?) AND TI=(causal inference or causal analysis or causal effect*))) AND ALL=(target trial or emulat* or target trial emulation)

For peer review only

For peer review only - http://bmjopen.bmj.com/site/about/guidelines.xhtml

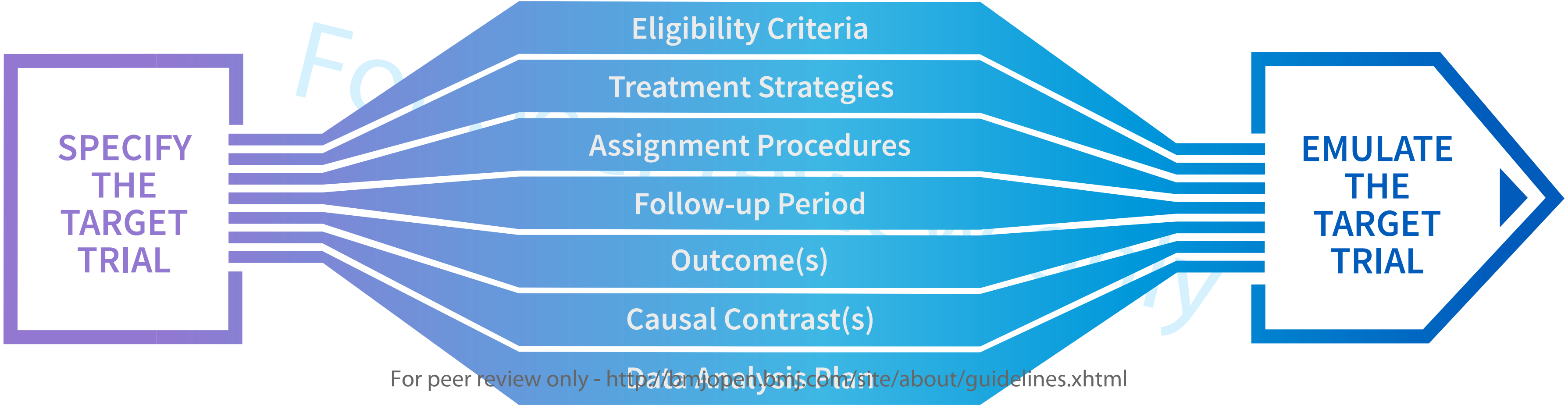
SPECIFY THE TARGET TRIAL
Eligibility Criteria
Treatment Strategies
Assignment Procedures
Follow-up Period
Outcome(s)
Causal Contrast(s)
Data Analysis Plan
EMULATE THE TARGET TRIAL

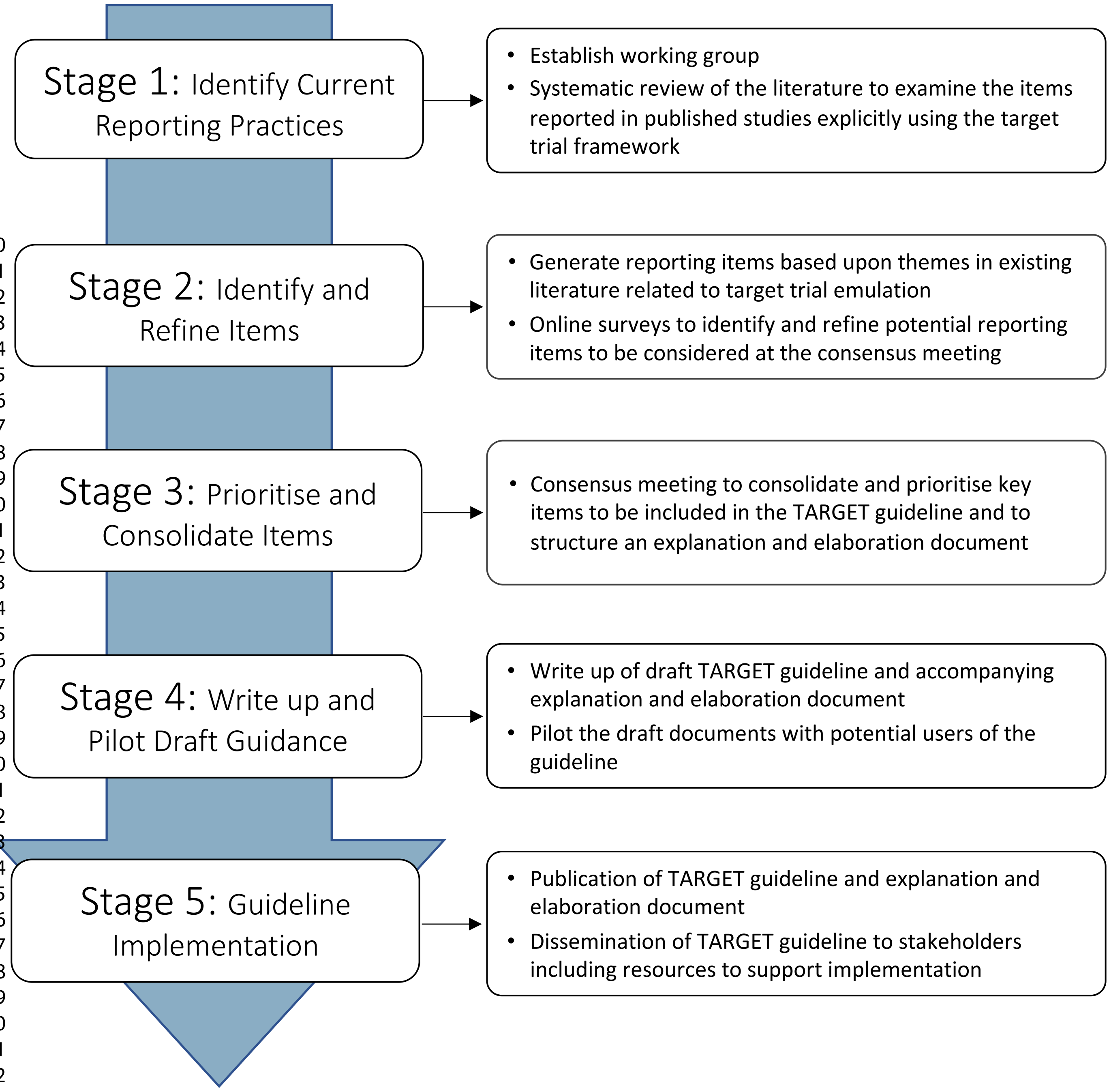
Stage 1: Identify Current Reporting Practices
• Establish working group
• Systematic review of the literature to examine the items reported in published studies explicitly using the target trial framework
Stage 2: Identify and Refine Items
• Generate reporting items based upon themes in existing literature related to target trial emulation
• Online surveys to identify and refine potential reporting items to be considered at the consensus meeting
Stage 3: Prioritise and Consolidate Items
• Consensus meeting to consolidate and prioritise key items to be included in the TARGET guideline and to structure an explanation and elaboration document
Stage 4: Write up and Pilot Draft Guidance
• Write up of draft TARGET guideline and accompanying explanation and elaboration document
• Pilot the draft documents with potential users of the guideline
Stage 5: Guideline Implementation
• Publication of TARGET guideline and explanation and elaboration document
• Dissemination of TARGET guideline to stakeholders including resources to support implementation

BMJ Open

Development of the Transparent Reporting of Observational Studies Emulating a Target Trial (TARGET) Guideline

Journal:	*BMJ Open*
Manuscript ID	bmjopen-2023-074626.R1
Article Type:	Protocol
Date Submitted by the Author:	26-Jul-2023

Complete List of Authors:	Hansford, Harrison; University of New South Wales; Neuroscience Research Australia<br>Cashin, Aidan; Neuroscience Research Australia, Centre for Pain IMPACT; University of New South Wales, School of Health Sciences, Faculty of Medicine and Health<br>Jones, Matthew; University of New South Wales; Neuroscience Research Australia, Centre for Pain IMPACT<br>Swanson, Sonja; University of Pittsburgh, Department of Epidemiology; Harvard T.H. Chan School of Public Health, CAUSALab, Department of Epidemiology<br>Islam, Nazrul; University of Oxford, Big data institute; University of Southampton, Faculty of Medicine<br>Dahabreh, Isa; , Beth Israel Deaconess Medical Center and Harvard Medical School, Richard A. and Susan F. Smith Center for Outcomes Research; Harvard T.H. Chan School of Public Health, CAUSALab, Department of Epidemiology, Department of Biostatistics<br>Dickerman, Barbra; Harvard TH Chan School of Public Health, CAUSALab; Harvard TH Chan School of Public Health, Department of Epidemiology<br>Egger, Matthias; University of Bern, Institute of Social & Preventive Medicine; University of Cape Town Faculty of Health Sciences, Centre for Infectious Disease Epidemiology and Research<br>Garcia-De-Albeniz, Xavier ; Harvard University T H Chan School of Public Health, CAUSALab; RTI Health Solutions Barcelona<br>Golub, Robert ; Northwestern University Feinberg School of Medicine<br>Lodi, Sara; Harvard TH Chan School of Public Health, CAUSALab; Boston University School of Public Health, Department of Biostatistics<br>Moreno-Betancur , Margarita ; Murdoch Children's Research Institute, Melbourne, Clinical Epidemiology & Biostatistics Unit; The University of Melbourne, Department of Paediatrics<br>Pearson, Sallie-Anne; University of New South Wales, School of Population Health<br>Schneeweiss , Sebastian ; Harvard Medical School, Boston, USA, Division of Pharmacoepidemiology, Department of Medicine, Brigham & Women's Hospital,<br>Sterne, Jonathan; University of Bristol, Department of Population Health Sciences; NIHR Bristol Biomedical Research Centre<br>Sharp, Melissa; RCSI University of Medicine and Health Sciences, Department of General Practice<br>Stuart, Elizabeth A; Johns Hopkins Bloomberg School of Public Health, Department of Mental Health

	Hernan, M; Harvard School of Public Health, CAUSALab; Harvard T.H Chan School of Public Health, Department of Epidemiology, Department of Biostatistics<br>Lee, Hopin; University of Exeter Medical School<br>McAuley, James; University of New South Wales, School of Health Sciences, Faculty of Medicine and Health; Neuroscience Research Australia, Centre for Pain IMPACT
**Primary Subject Heading**:	Epidemiology
Secondary Subject Heading:	Research methods
Keywords:	EPIDEMIOLOGY, Retrospective Studies, STATISTICS & RESEARCH METHODS

Development of the Transparent Reporting of Observational Studies Emulating a Target Trial (TARGET) Guideline

Harrison J. Hansford[1,2], Aidan G. Cashin[1,2], Matthew D. Jones[1,2], Sonja A. Swanson[3,8,9], Nazrul Islam[5,6], Issa J. Dahabreh[7,8, 9,10], Barbra A. Dickerman[8,9], Matthias Egger[11,12,13], Xabier Garcia-Albeniz[8, 14], Robert M. Golub[15], Sara Lodi[8,16], Margarita Moreno-Betancur[17,18], Sallie-Anne Pearson[19], Sebastian Schneeweiss[20], Jonathan A. C. Sterne[21,22,23], Melissa K. Sharp[24], Elizabeth A. Stuart[25], Miguel A. Hernán[8,9,10], Hopin Lee[26], James H. McAuley[1,2]

1. School of Health Sciences, Faculty of Medicine and Health, University of New South Wales, Sydney, Australia
2. Centre for Pain IMPACT, Neuroscience Research Australia, Sydney, Australia
3. Department of Epidemiology, University of Pittsburgh, Pittsburgh, United States of America
5. Oxford Population Health, Big Data Institute, University of Oxford, Oxford, UK
6. Faculty of Medicine, University of Southampton, Southampton, UK
7. Richard A. and Susan F. Smith Center for Outcomes Research, Beth Israel Deaconess Medical Center and Harvard Medical School, Boston, MA, USA
8. CAUSALab, Harvard T.H. Chan School of Public Health, Boston, MA, USA
9. Department of Epidemiology, Harvard T.H. Chan School of Public Health, Boston, MA, USA
10. Department of Biostatistics, Harvard T.H. Chan School of Public Health, Boston, MA, USA
11. Institute of Social and Preventive Medicine, University of Bern, Bern, Switzerland
12. Centre for Infectious Disease Epidemiology and Research, Faculty of Health Sciences, University of Cape Town, Cape Town, South Africa
13. Population Health Sciences, Bristol Medical School, University of Bristol, Bristol, UK
14. RTI Health Solutions, Barcelona, Spain
15. Northwestern University Feinberg School of Medicine, Chicago, IL, USA
16. Department of Biostatistics, Boston University School of Public Health, Boston, MA, USA
17. Clinical Epidemiology & Biostatistics Unit, Murdoch Children's Research Institute, Royal Children's Hospital, 50 Flemington Rd, Parkville, Melbourne, VIC, Australia
18. Department of Paediatrics, The University of Melbourne, Parkville, Australia

For peer review only

19. School of Population Health, Faculty of Medicine and Health, UNSW Sydney, Sydney, Australia

20. Division of Pharmacoepidemiology, Department of Medicine, Brigham & Women's Hospital, Harvard Medical School, Boston, MA, USA

21. Department of Population Health Sciences, Bristol Medical School, University of Bristol, UK

22. NIHR Bristol Biomedical Research Centre, UK

23. Health Data Research UK South-West, Bristol, UK

24. Department of General Practice, RCSI University of Medicine and Health Sciences, Dublin, Ireland

25. Department of Mental Health, Johns Hopkins Bloomberg School of Public Health, Baltimore, MD, USA

26. University of Exeter Medical School, Exeter, UK

Corresponding Author

Harrison J Hansford

E: h.hansford@unsw.edu.au

School of Health Sciences, UNSW Sydney,

2052, Sydney, Australia

Words: 2809

For peer review only

Abstract

Background

Observational studies are increasingly used to inform health decision-making when randomised trials are not feasible, ethical, or timely. The target trial approach provides a framework to help minimise common biases in observational studies that aim to estimate the causal effect of interventions. Incomplete reporting of studies using the target trial framework limits the ability for clinicians, researchers, patients, and other decision-makers to appraise, synthesise, and interpret findings to inform clinical and public health practice and policy. This paper describes the methods that we will use to develop the transparent reporting of observational studies emulating a target trial (TARGET) reporting guideline.

Methods/design

The TARGET reporting guideline will be developed in five stages following recommended guidance. The first stage will identify target trial reporting practices by systematically reviewing published studies that explicitly emulated a target trial. The second stage will identify and refine items to be considered for inclusion in the TARGET guideline by consulting content experts using online surveys. The third stage will prioritise and consolidate key items to be included in the TARGET guideline at a consensus meeting of TARGET investigators. The fourth stage will produce and pilot-test the TARGET guideline and explanation and elaboration document with relevant

For peer review only

For peer review only - http://bmjopen.bmj.com/site/about/guidelines.xhtml

stakeholders. The fifth stage will disseminate the TARGET guideline and resources via journals, conferences, and courses.

Ethics and Dissemination

Ethical approval for the survey to be conducted has been attained (HC220536). The TARGET guideline will be disseminated widely in partnership with stakeholders to maximise adoption and improve reporting of these studies.

Key words: target trial emulation, causal inference, reporting guideline, observational studies

Strengths and Limitations

- The TARGET reporting guideline will be developed according to recommendations for health research reporting guidelines
- The TARGET working group has been established to include stakeholders from a variety of backgrounds
- A comprehensive piloting phase may increase the usability and uptake of the reporting guideline

For peer review only

Introduction

Observational studies can provide evidence on the causal effects of interventions when it is not feasible, ethical, or timely to conduct a relevant randomised trial. However, making causal inferences from observational data is challenging due to confounding and design-related biases such as selection bias and immortal time bias.[1 2] Design-related biases can be avoided using the target trial framework.[3 4] The framework involves the specification of the hypothetical randomised pragmatic trial — the target trial — that would ideally be conducted and how this trial might be emulated using observational data.[3 4] The two stages of the target trial framework are 1) specification of the target trial, and 2) emulation of the target trial.[3 4] Using observational data to mimic a randomised experiment was proposed in the mid 20th century,[5-8] and extended to time-varying treatments by Robins in 1986.[9]

The value of using the target trial framework to design the analysis of observational studies has been recognised by international regulatory bodies in the field of medicine and health,[10-14] and the framework underpins the widely-used ROBINS-I tool for assessing risk of bias in non-randomised studies of interventions.[15] Studies that are explicit in using the target trial framework have been published with increasing frequency in leading general medical and specialty journals.[16-21]

Application of the target trial framework requires the complete specification of the target trial protocol and its emulation (Figure 1).[3] Hernán & Robins[3] provide a template

For peer review only - http://bmjopen.bmj.com/site/about/guidelines.xhtml

for specifying a target trial and its emulation; however, there is currently no detailed guidance on reporting a study designed to emulate a target trial. Incomplete reporting of these studies limits the ability of clinicians, researchers, patients, and other decision-makers to appraise and synthesise findings or interpret them to inform clinical and public health practice and policy. A reporting guideline that expands upon the initial target trial emulation template[3] is needed to provide authors with comprehensive recommendations on how to completely and transparently report a study emulating a target trial.

[INSERT FIGURE 1]

To address this gap, we outline the processes and methods that used to develop a reporting guideline for studies emulating a target trial – TARGET (Transparent reporting of observational studies emulating a target trial).

Objective

The objective of the TARGET guideline is to provide guidance on the minimum set of items that should be reported to provide a clear and transparent account of observational studies that investigate the comparative effectiveness and safety of health interventions explicitly using the target trial framework.

For peer review only

Methods

We will develop the TARGET Guideline in five stages following recommendations for the development of health research reporting guidelines (Figure 2).[22] The start date for the study was late 2022, with the planned end date early 2025.

[INSERT FIGURE 2]

For peer review only

TARGET working group

The TARGET working group is made up of the steering committee and project team (Supplementary Material 1). The group was established to collate expertise on target trial emulation methodology, epidemiology, clinical trials, biostatistics, reporting guideline development, and knowledge of regulatory and journal editorial processes. The working group will oversee recruitment of participants for Stages 2 and 3 and contribute to writing and disseminating the guideline documents.

Stage 1: Identify current reporting practices

The systematic review aims to assess whether and how important items are reported by published studies explicitly emulating a target trial and whether reporting guidance (e.g., STROBE[23]) was used. The protocol for this systematic review was registered on the Open Science Framework on 13 March 2022 (osf.io/uj56m).

Databases, eligibility, and search terms

We will search Medline, EMBASE, PsycINFO and Science Citation Index for observational studies that stated in their methods that they explicitly emulated a target trial. We will exclude studies not written in English, not in the field of medicine and health, not conducted in humans, or not observational designs. Many observational studies may implicitly use the framework of a randomised trial. However, to be included in this review studies must be explicit in their attempt to emulate a target trial (e.g., stated 'target trial emulation' in the article). To identify eligible studies, we developed a literature search in collaboration with an expert librarian at the University of Oxford. Our approach used sensitive search terms including emulat*, target trial, observational data, real-world data, comparative effectiveness, and causal inference, to try to capture all papers explicitly emulating a target trial. The complete search strategy is in Supplementary Material 2. We will conduct forward citation tracking of selected seminal articles to maximise the chance of retrieving all relevant articles.[3 9 24-26] We will also include papers known to the authorship team. In duplicate, independent reviewers will conduct title, abstract, and full text screening. We will resolve disagreements between reviewers through discussion.

Data Extraction

We will extract items regarded by the steering committee as potentially important for the reporting of a target trial emulation, including those outlined by Hernán and Robins, 2016.[3] Two independent reviewers will extract information on study authors, year of publication, journal, sub-field of medicine, study design, sample size, intervention,

For peer review only - http://bmjopen.bmj.com/site/about/guidelines.xhtml

comparison group, outcomes assessed, and whether the study was prospectively registered. We will extract items relevant to the methods and results of the target trial emulation, including whether and how all components of the protocol of the proposed target trial, and how they were emulated, were specified (i.e., eligibility criteria, treatment strategies, assignment procedures, follow-up period, outcome(s), causal contrast(s), and data analysis plan). We will enter data into a standardised data extraction form which two authors will pilot with a selection of included studies. We will resolve disagreements in data extraction between reviewers through discussion, or where necessary, consultation with a third reviewer.

Data analysis

We will use R[27] for all data analyses. Categorical variables will be summarised using frequencies and percentages. Continuous variables will be summarised using mean and standard deviation, or median and interquartile range, as appropriate.

Outcomes of the systematic review

The systematic review will provide evidence on reporting in studies explicitly emulating a target trial. We acknowledge that excluding studies not written in English and unpublished studies may cause potentially relevant articles to be excluded. The findings will inform the online surveys (Stage 2) and the consensus meeting (Stage 3). We will submit the findings of this review for publication and all data and code made publicly available.

For peer review only - http://bmjopen.bmj.com/site/about/guidelines.xhtml

Stage 2: Identify and refine items for the TARGET guideline

We will conduct two online surveys to generate a list of candidate items that add detail to each of the protocol elements in Figure 1.

Ethics

Ethical approval has been obtained for the online surveys from the University of New South Wales Human Research Ethics Committee (HC220536).

Selection of initial items

The steering group will develop a list of key items, informed by the systematic review (Stage 1), and the target trial framework described by Hernán & Robins,[3] thought important for the conduct and reporting target trial emulations (Figure 1). Other potential sources of items include: published guidance for observational studies and randomised controlled trials, the ROBINS-I tool,[15] and studies that describe items that may be important for the conduct or reporting of target trial emulations.

Participants

Members of the TARGET working group (Supplementary Material 1) will be invited to participate in the surveys.

Procedure

For peer review only

We will host two online surveys using REDCap.[28 29] We will send each online survey via email to the participants. We will ask participants to rate the importance of each potential reporting item on a 9-point Likert scale (1, “not important”, to 9, “critically important”). Participants will have the opportunity to provide suggestions or modifications to the wording of items as well as suggest additional items or make other comments.

In the second survey, we will send participants a summary of the results for each potential reporting item (mean scores and standard deviations, median scores and interquartile ranges, and histograms), their own score for each item, and any comments from participants on each item from the first survey. We will also present any new items and suggested modifications to items. We will then invite participants to re-score the importance of each item, and score any additional items, considering the aggregated ratings. Participants will have the opportunity to provide additional feedback on each item in the form of open ended responses.

Analysis

Continuous variables will be summarised using mean and standard deviation, or median and interquartile range, as appropriate. We will analyse the free-text responses from the first and second surveys using an inductive approach,[30] in which we will use reflexive thematic[30] analysis to identify, organise and generate codes, and then identify themes found within the dataset. Briefly, inductive coding is a process

For peer review only

pooling common ideas without trying to fit ideas/codes into a pre-existing framework. These data will contribute to the creation of new items and modification of existing items to be included in the subsequent survey.

Outcome of the online surveys

We will generate a preliminary list of items with corresponding ratings of importance to be considered in the TARGET guideline at the consensus meeting (Stage 3). We will also generate qualitative insights to guide item refinement and prioritisation in preparation for the consensus meeting.

Stage 3 – Consolidate and prioritise key items to be included in the TARGET guideline

A consensus meeting will finalise reporting items for the TARGET guideline. [22] The consensus meeting will follow suggested methods for developing reporting guidelines,[22] including guidance for consensus-based methods currently being developed which we will use if they become available.[31]

Process

We will invite stakeholders identified by the working group to participate in a two-day consensus meeting. The TARGET working group will ensure that the expertise of consensus meeting participants includes target trial emulation methodology, epidemiology, clinical trials, biostatistics, reporting guideline development, and regulatory and journal editorial processes. Prior to the consensus meeting, the core

For peer review only - http://bmjopen.bmj.com/site/about/guidelines.xhtml

team will provide attendees with evidence from the systematic review (Stage 1) and findings from the online surveys (Stage 2) including a draft of the items proposed for inclusion in the guideline. We will present the findings from Stage 1 and 2 at the consensus meeting. A member of the TARGET working group will facilitate a structured discussion on the rationale for including items from the online surveys. If there are disagreements, they will first be debated and, if disagreements remain, we will hold an anonymised vote to establish the importance of including the item in the guideline. For the anonymised vote, a simple majority will be sufficient to guide the inclusion/exclusion of an item. The meeting will conclude with discussion about the content and production of relevant documents (TARGET guideline, draft explanation and elaboration document) as well as strategies for dissemination and implementation. Following the conclusion of the consensus meeting, we will circulate a report on the outcome to the meeting participants for review and approval.

Stage 4 – Development and piloting of the draft TARGET guideline and explanation and elaboration document

Stage 4 involves drafting the TARGET guideline and accompanying explanation and elaboration document to ensure that the wording and content of the documents are clear, precise, and suitable for all identified stakeholders. The purpose of the explanation and elaboration document is to explain each item by providing background information, a rationale, and clear reporting examples from published target trial emulations. We will design the explanation and elaboration document to facilitate

For peer review only

adherence to the TARGET guideline by clarifying the importance of each item, highlighting relevant reporting issues and providing examples to assist authors using the guideline. The consensus meeting participants may be asked to review and comment on the draft TARGET guideline and explanation and elaboration document.

We will evaluate the TARGET guideline by piloting the proposed guideline and the explanation and elaboration document with 20-30 expert methodologists and potential users of TARGET, identified from TARGET working group networks. We will ask participants to provide general feedback on accessibility and usability, and to identify possible reporting items that might have been overlooked. We will also ask for specific feedback about the utility and clarity of each TARGET item. We will collect data through online surveys, hosted by REDCap.[28 29] We will incorporate feedback from the piloting exercise into the final guideline and explanation and elaboration document, as required. If suggested revisions are extensive, we will conduct a further round of piloting.

Patient and public involvement

Potential users of this research include health researchers conducting observational analyses, regulatory bodies, public health and other health decision-makers. We aim to include relevant decision-makers in the piloting phase of the guideline development process to maximise the usefulness and uptake of the TARGET guideline. Participants

For peer review only - http://bmjopen.bmj.com/site/about/guidelines.xhtml

in any stage of the guideline development will be informed of the results and final guidance.

Stage 5 – Guideline implementation

The goal of the final stage of guideline development is to maximise reach and use of the TARGET guideline. The TARGET working group will guide the dissemination strategy with advice from consensus meeting participants. We aim to publish the TARGET guideline and the explanation and elaboration document and disseminate the findings through traditional and social media. We will engage journal editors and funding agencies to encourage TARGET guideline endorsement alongside other published reporting guidance. We will publicly host the TARGET guideline and explanation and elaboration paper, and any other relevant material on a TARGET website. We will index the guideline on the Enhancing the QUAlity and Transparency Of health Research (EQUATOR) Network website.[32 33] We will create online resources including infographics, blog posts and podcasts, which will be available on the TARGET website. We will share the TARGET guideline with authors in the field, and at relevant scientific conferences and methodological courses.

For peer review only

Declarations

Ethics approval and consent to participate

Not Applicable

Consent for publication

All authors consent to publication of this manuscript

Availability of data and materials

Not applicable

Funding

There was no specific funding for this study. HJH was supported by an Australian National Health and Medical Research Council (NHMRC) Postgraduate Scholarship, a PhD Top-Up Scholarship from Neuroscience Research Australia, and was a Neuroscience Research Australia PhD Pearl sponsored by Paul Salteri AO. AGC was supported by an Australian NHMRC Investigator Grant (ID 2010088). MM-B was supported by an Australian NHMRC Investigator Grant (ID 2009572). JMH was supported by an Australian NHMRC Investigator Grant (ID 2010128). BAD was supported by a grant from the National Institutes of Health (R00 CA248335). ME was supported by grants from the National Institutes of Health (R01 AI152772-01, 5U01-AI069924-05) and the Swiss National Science Foundation (32FP30-174281). NI was

For peer review only

For peer review only - http://bmjopen.bmj.com/site/about/guidelines.xhtml

supported by grants from the National Institute for Health and Care Research (HDRUK2022.0313) and the UK Office for National Statistics (2002563). MAH was supported by NIH grant R37 AI102634.

Competing interests

All authors declare no competing interests.

Author Contributions

HJH, AGC, MDJ, HL, JHM, conceived the idea for the project protocol. All authors contributed to the design and methodology of the project protocol. HJH and AGC wrote the first draft of the manuscript. MAH, SAS, IJD, BAD, XG-A, ME, RMG, NI, SL, MM-B, SAP, SS, JACS, MKS, EAS provided feedback, revised the manuscript and have read and approved the final version.

Acknowledgements

We acknowledge Nia Roberts, outreach librarian and information specialist at the University of Oxford for assistance designing the literature search.

For peer review only

Abbreviations

EQUATOR: Enhancing the QUAlity and Transparency Of health Research

REDCap: Research Electronic Data Capture

STROBE: Strengthening the Reporting of Observational Studies in Epidemiology

TARGET: TrAnsparent ReportinG of studies Emulating a Target trial

Figure Captions

Figure 1: Elements relevant to both the specification and emulation of the target trial described by Hernán & Robins[3]

Figure 2: Workflow for the development of the TARGET guideline

For peer review only

References

1. Hernán MA, Sauer BC, Hernández-Díaz S, et al. Specifying a target trial prevents immortal time bias and other self-inflicted injuries in observational analyses. *J Clin Epidemiol* 2016;79:70-75. doi: 10.1016/j.jclinepi.2016.04.014 [published Online First: 2016/05/31]

2. Dickerman BA, García-Albéniz X, Logan RW, et al. Avoidable flaws in observational analyses: an application to statins and cancer. *Nature Medicine* 2019;25(10):1601-06. doi: 10.1038/s41591-019-0597-x

3. Hernán MA, Robins JM. Using big data to emulate a target trial when a randomized trial is not available. *American journal of epidemiology* 2016;183(8):758-64.

4. Hernán MA. Methods of public health research—strengthening causal inference from observational data. *New England Journal of Medicine* 2021;385(15):1345-48.

5. Cochran WG. Observational studies. *Statistical papers in honor of George W Snedecor* 1972:77-90.

6. Dorn HF. Philosophy of inferences from retrospective studies. *American Journal of Public Health and the Nations Health* 1953;43(6_Pt_1):677-83.

7. Rubin DB. Estimating causal effects of treatments in randomized and nonrandomized studies. *Journal of Educational Psychology* 1974;66(5):688.

8. Wold H. Causality and econometrics. *Econometrica: Journal of the Econometric Society* 1954:162-77.

9. Robins J. A new approach to causal inference in mortality studies with a sustained exposure period—application to control of the healthy worker survivor effect. *Mathematical Modelling* 1986;7(9-12):1393-512.

10. Concato J, Stein P, Dal Pan GJ, et al. Randomized, observational, interventional, and real-world—What's in a name? *Pharmacoepidemiology and Drug Safety* 2020;29(11):1514-17.

11. Agency EM. European medicines agencies network strategy to 2025. The Netherlands: Health of Medicines Agencies, 2020.

12. Excellence NIfHaC. The NICE strategy 2021 to 2026, 2021.

13. Health Canada. Optimizing the Use of Real World Evidence to Inform Regulatory Decision-Making: Government of Canada, 2019.

14. Therapeutic Goods Administration. Real world evidence and patient reported outcomes in the regulatory context. https://www.tga.gov.au/review-real-world-evidence-and-patient-reported-outcomes: Australian Government, Department of Health, 2021.

15. Sterne JA, Hernán MA, Reeves BC, et al. ROBINS-I: a tool for assessing risk of bias in non-randomised studies of interventions. *BMJ* 2016;355:i4919. doi: 10.1136/bmj.i4919

16. Garcia-Albeniz XH, J.: Bretthauer, M.: Hernan, M. A. Effectiveness of screening colonoscopy to prevent colorectal cancer among medicare beneficiaries aged 70 to 79 years: A prospective observational study. *Annals of Internal Medicine* 2017 doi: http://dx.doi.org/10.7326/M16-0758

17. Mahévas M, Tran V-T, Roumier M, et al. Clinical efficacy of hydroxychloroquine in patients with covid-19 pneumonia who require oxygen: observational comparative study using routine care data. *BMJ* 2020;369:m1844. doi: 10.1136/bmj.m1844

18. Kaura A, Sterne JAC, Trickey A, et al. Invasive versus non-invasive management of older patients with non-ST elevation myocardial infarction (SENIOR-NSTEMI): a cohort

For peer review only

For peer review only - http://bmjopen.bmj.com/site/about/guidelines.xhtml

study based on routine clinical data. *The Lancet* 2020;396(10251):623-34. doi: 10.1016/S0140-6736(20)30930-2

19. Emilsson L, García-Albéniz X, Logan RW, et al. Examining Bias in Studies of Statin Treatment and Survival in Patients With Cancer. *JAMA Oncol* 2018;4(1):63-70. doi: 10.1001/jamaoncol.2017.2752
20. Chan You S, Krumholz HM, Suchard MA, et al. Comprehensive Comparative Effectiveness and Safety of First-Line β-Blocker Monotherapy in Hypertensive Patients: A Large-Scale Multicenter Observational Study. *Hypertension* 2021;77(5):1528-38. doi: 10.1161/hypertensionaha.120.16402 [published Online First: 20210329]
21. Caniglia EC, Robins JM, Cain LE, et al. Emulating a trial of joint dynamic strategies: An application to monitoring and treatment of HIV-positive individuals. *Stat Med* 2019;38(13):2428-46. doi: 10.1002/sim.8120 [published Online First: 20190318]
22. Moher D, Schulz KF, Simera I, et al. Guidance for developers of health research reporting guidelines. *PLoS medicine* 2010;7(2):e1000217.
23. Von Elm E, Altman DG, Egger M, et al. The Strengthening the Reporting of Observational Studies in Epidemiology (STROBE) statement: guidelines for reporting observational studies. *Bulletin of the World Health Organization* 2007;85:867-72.
24. Hernán MA, Sauer BC, Hernández-Díaz S, et al. Specifying a target trial prevents immortal time bias and other self-inflicted injuries in observational analyses. *Journal of Clinical Epidemiology* 2016;79:70-75. doi: 10.1016/j.jclinepi.2016.04.014 [published Online First: 2016/05/31]
25. Hernán MA. How to estimate the effect of treatment duration on survival outcomes using observational data. *Bmj* 2018;360:k182. doi: 10.1136/bmj.k182 [published Online First: 2018/02/09]
26. Robins JM, Hernan, M. J. Estimation of the causal effects of time-varying exposures. In: Fitzmaurice G, Davidian M, Verbeke G, et al., eds. Longitudinal Data Analysis: CRC press 2008.
27. R Core Team. R: A language and environment for statistical computing. 2013
28. Harris PA, Taylor R, Minor BL, et al. The REDCap consortium: Building an international community of software platform partners. *Journal of Biomedical Informatics* 2019;95:103208. doi: https://doi.org/10.1016/j.jbi.2019.103208
29. Harris PA, Taylor R, Thielke R, et al. Research electronic data capture (REDCap)—A metadata-driven methodology and workflow process for providing translational research informatics support. *Journal of Biomedical Informatics* 2009;42(2):377-81. doi: https://doi.org/10.1016/j.jbi.2008.08.010
30. Braun V, Clarke V. Using thematic analysis in psychology. *Qualitative Research in Psychology* 2006;3(2):77-101. doi: 10.1191/1478088706qp063oa
31. Gattrell WT, Hungin AP, Price A, et al. ACCORD guideline for reporting consensus-based methods in biomedical research and clinical practice: a study protocol. *Research Integrity and Peer Review* 2022;7(1):3. doi: 10.1186/s41073-022-00122-0
32. The EQUATOR Network. EQUATOR Network - Enhancing the QUAlity and Transparency Of health Research 2022 [Available from: https://www.equator-network.org/2022.
33. Simera I, Moher D, Hoey J, et al. A catalogue of reporting guidelines for health research. *European journal of clinical investigation* 2010;40(1):35-53.

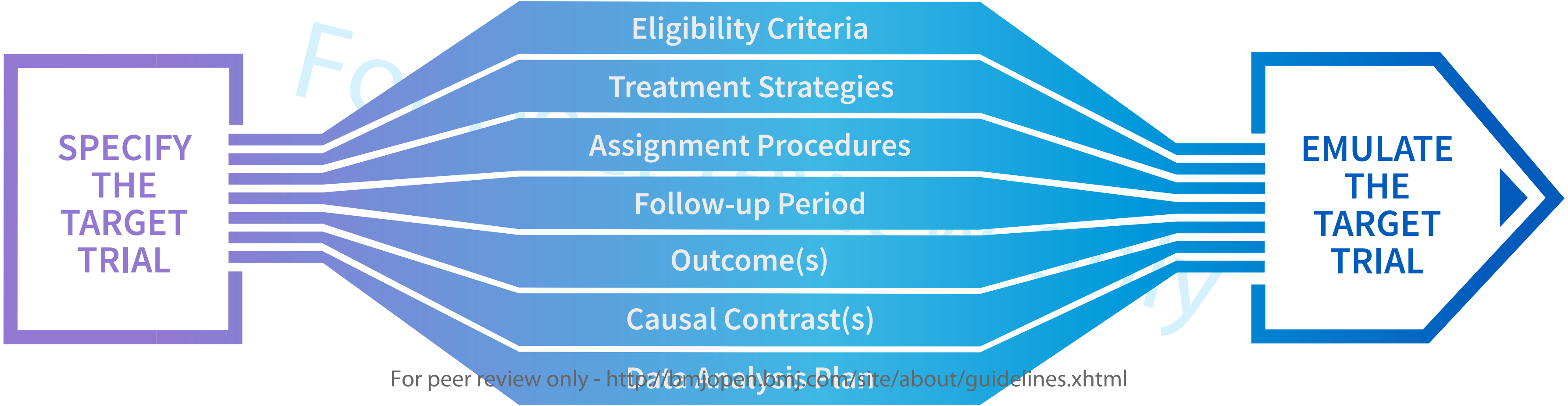

For peer review only - http://bmjopen.bmj.com/site/about/guidelines.xhtml

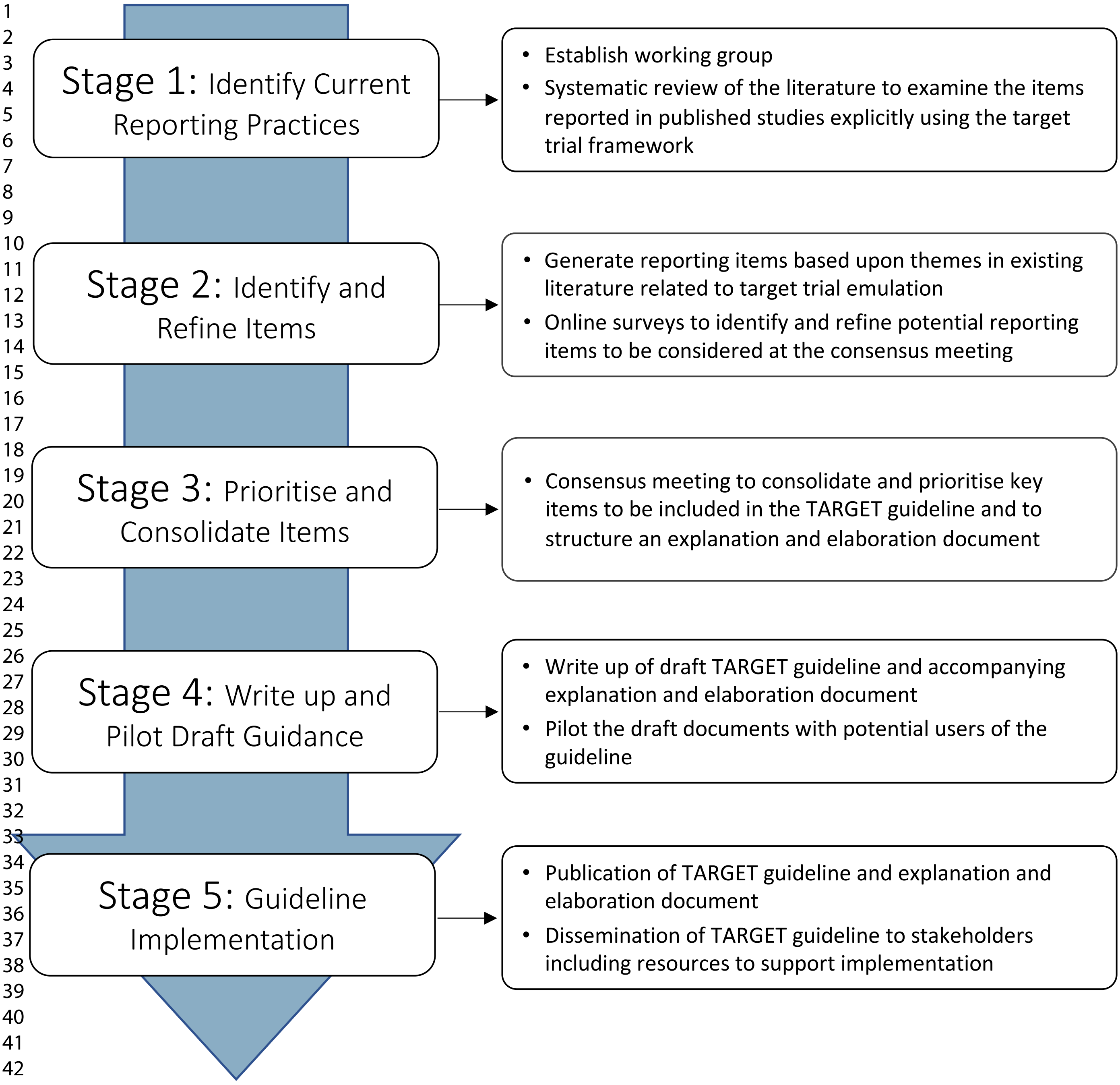
Stage 1: Identify Current Reporting Practices
Establish working group
Systematic review of the literature to examine the items reported in published studies explicitly using the target trial framework
Stage 2: Identify and Refine Items
Generate reporting items based upon themes in existing literature related to target trial emulation
Online surveys to identify and refine potential reporting items to be considered at the consensus meeting
Stage 3: Prioritise and Consolidate Items
Consensus meeting to consolidate and prioritise key items to be included in the TARGET guideline and to structure an explanation and elaboration document
Stage 4: Write up and Pilot Draft Guidance
Write up of draft TARGET guideline and accompanying explanation and elaboration document
Pilot the draft documents with potential users of the guideline
Stage 5: Guideline Implementation
Publication of TARGET guideline and explanation and elaboration document
Dissemination of TARGET guideline to stakeholders including resources to support implementation

Supplementary Material

Supplementary Material 1: TARGET working group members (alphabetical)

Steering committee
Dr Aidan G. Cashin
Mr Harrison J. Hansford
Prof Miguel A. Hernán
Dr Hopin Lee
Dr Matthew D. Jones
Prof James H. McAuley
A/Prof Sonja A. Swanson

Project team
A/Prof Issa J. Dahabreh
A/Prof Barbra A. Dickerman
Prof Matthias Egger
Dr Xabier Garcia-Albeniz
Prof Robert M. Golub
A/Prof Nazrul Islam
A/Prof Sara Lodi
A/Prof Margarita Moreno-Betancur
Prof Sallie A. Pearson
Prof Sebastian Schneeweiss
Prof Jonathan A. C. Sterne
Dr Melissa K. Sharp
Prof Elizabeth A. Stuart

For peer review only

Supplementary Material 2: Complete search strategies for all databases

Medline

1 (emulat* adj5 trial?).mp.
2 (target adj (trial? or experiment?)).mp.
3. (observational adj (stud* or research or data)).mp.
4. ((real world or rwd) adj2 (stud* or research or data)).mp.
5. (routine* adj2 data).mp.
6. (comparative effectiveness adj2 (stud* or research or data)).mp.
7. (emulat* or propensity score? or (causal adj2 (inference? or analys?s or effect*))).mp.
8. 3 or 4 or 5 or 6 or 7
9. 2 and 8
10. (target adj (trial? or experiment?)).ti.
11. 1 or 9 or 10
Filtered for time (2012-2022) manually after search

Embase

1. (emulat* adj5 trial?).mp.
2. (target adj (trial? or experiment?)).mp.
3. (observational adj (stud* or research or data)).mp.
4. ((real world or rwd) adj2 (stud* or research or data)).mp.
5. (routine* adj2 data).mp.
6. (comparative effectiveness adj2 (stud* or research or data)).mp.
7. (emulat* or propensity score? or (causal adj2 (inference? or analys?s or effect*))).mp.
8. 3 or 4 or 5 or 6 or 7
9. 2 and 8
10. (target adj (trial? or experiment?)).ti.
11. 1 or 9 or 10

psycINFO

noft(target trial emulat*) OR ((noft(real world data) OR (noft(emulat* trial)) OR noft(observational) OR noft(routine* data)) AND noft(comparative effective*) AND noft(causal infer*))

Web of Science

(TI=(emulat* trial)) OR (TI=(real world data) OR TI=(routine* data) OR TI=(comparative effectiveness study comparative effectiveness research or comparative effectiveness data) OR (TI=(emulat* or propensity score?) AND TI=(causal inference or causal analysis or causal effect*))) AND ALL=(target trial or emulat* or target trial emulation)

For peer review only

BMJ Open

Development of the Transparent Reporting of Observational Studies Emulating a Target Trial (TARGET) Guideline

Journal:	*BMJ Open*
Manuscript ID	bmjopen-2023-074626.R2
Article Type:	Protocol
Date Submitted by the Author:	24-Aug-2023

Complete List of Authors:	Hansford, Harrison; University of New South Wales; Neuroscience Research Australia<br>Cashin, Aidan; Neuroscience Research Australia, Centre for Pain IMPACT; University of New South Wales, School of Health Sciences, Faculty of Medicine and Health<br>Jones, Matthew; University of New South Wales; Neuroscience Research Australia, Centre for Pain IMPACT<br>Swanson, Sonja; University of Pittsburgh, Department of Epidemiology; Harvard T.H. Chan School of Public Health, CAUSALab, Department of Epidemiology<br>Islam, Nazrul; University of Oxford, Big data institute; University of Southampton, Faculty of Medicine<br>Dahabreh, Isa; , Beth Israel Deaconess Medical Center and Harvard Medical School, Richard A. and Susan F. Smith Center for Outcomes Research; Harvard T.H. Chan School of Public Health, CAUSALab, Department of Epidemiology, Department of Biostatistics<br>Dickerman, Barbra; Harvard TH Chan School of Public Health, CAUSALab; Harvard TH Chan School of Public Health, Department of Epidemiology<br>Egger, Matthias; University of Bern, Institute of Social & Preventive Medicine; University of Cape Town Faculty of Health Sciences, Centre for Infectious Disease Epidemiology and Research<br>Garcia-De-Albeniz, Xavier ; Harvard University T H Chan School of Public Health, CAUSALab; RTI Health Solutions Barcelona<br>Golub, Robert ; Northwestern University Feinberg School of Medicine<br>Lodi, Sara; Harvard TH Chan School of Public Health, CAUSALab; Boston University School of Public Health, Department of Biostatistics<br>Moreno-Betancur , Margarita ; Murdoch Children's Research Institute, Melbourne, Clinical Epidemiology & Biostatistics Unit; The University of Melbourne, Department of Paediatrics<br>Pearson, Sallie-Anne; University of New South Wales, School of Population Health<br>Schneeweiss , Sebastian ; Harvard Medical School, Boston, USA, Division of Pharmacoepidemiology, Department of Medicine, Brigham & Women's Hospital,<br>Sterne, Jonathan; University of Bristol, Department of Population Health Sciences; NIHR Bristol Biomedical Research Centre<br>Sharp, Melissa; RCSI University of Medicine and Health Sciences, Department of General Practice<br>Stuart, Elizabeth A; Johns Hopkins Bloomberg School of Public Health, Department of Mental Health

	Hernan, M; Harvard School of Public Health, CAUSALab; Harvard T.H Chan School of Public Health, Department of Epidemiology, Department of Biostatistics<br>Lee, Hopin; University of Exeter Medical School<br>McAuley, James; University of New South Wales, School of Health Sciences, Faculty of Medicine and Health; Neuroscience Research Australia, Centre for Pain IMPACT

**Primary Subject Heading**:	Epidemiology
Secondary Subject Heading:	Research methods
Keywords:	EPIDEMIOLOGY, Retrospective Studies, STATISTICS & RESEARCH METHODS

SCHOLARONE™ Manuscripts

Development of the Transparent Reporting of Observational Studies Emulating a Target Trial (TARGET) Guideline

Harrison J. Hansford[1,2], Aidan G. Cashin[1,2], Matthew D. Jones[1,2], Sonja A. Swanson[3,8,9], Nazrul Islam[5,6], Issa J. Dahabreh[7,8, 9,10], Barbra A. Dickerman[8,9], Matthias Egger[11,12,13], Xabier Garcia-Albeniz[8, 14], Robert M. Golub[15], Sara Lodi[8,16], Margarita Moreno-Betancur[17,18], Sallie-Anne Pearson[19], Sebastian Schneeweiss[20], Jonathan A. C. Sterne[21,22,23], Melissa K. Sharp[24], Elizabeth A. Stuart[25], Miguel A. Hernán[8,9,10], Hopin Lee[26], James H. McAuley[1,2]

1. School of Health Sciences, Faculty of Medicine and Health, University of New South Wales, Sydney, Australia
2. Centre for Pain IMPACT, Neuroscience Research Australia, Sydney, Australia
3. Department of Epidemiology, University of Pittsburgh, Pittsburgh, United States of America
5. Oxford Population Health, Big Data Institute, University of Oxford, Oxford, UK
6. Faculty of Medicine, University of Southampton, Southampton, UK
7. Richard A. and Susan F. Smith Center for Outcomes Research, Beth Israel Deaconess Medical Center and Harvard Medical School, Boston, MA, USA
8. CAUSALab, Harvard T.H. Chan School of Public Health, Boston, MA, USA
9. Department of Epidemiology, Harvard T.H. Chan School of Public Health, Boston, MA, USA
10. Department of Biostatistics, Harvard T.H. Chan School of Public Health, Boston, MA, USA
11. Institute of Social and Preventive Medicine, University of Bern, Bern, Switzerland
12. Centre for Infectious Disease Epidemiology and Research, Faculty of Health Sciences, University of Cape Town, Cape Town, South Africa
13. Population Health Sciences, Bristol Medical School, University of Bristol, Bristol, UK
14. RTI Health Solutions, Barcelona, Spain
15. Northwestern University Feinberg School of Medicine, Chicago, IL, USA
16. Department of Biostatistics, Boston University School of Public Health, Boston, MA, USA
17. Clinical Epidemiology & Biostatistics Unit, Murdoch Children's Research Institute, Royal Children's Hospital, 50 Flemington Rd, Parkville, Melbourne, VIC, Australia
18. Department of Paediatrics, The University of Melbourne, Parkville, Australia

For peer review only

For peer review only - http://bmjopen.bmj.com/site/about/guidelines.xhtml

19. School of Population Health, Faculty of Medicine and Health, UNSW Sydney, Sydney, Australia
20. Division of Pharmacoepidemiology, Department of Medicine, Brigham & Women's Hospital, Harvard Medical School, Boston, MA, USA
21. Department of Population Health Sciences, Bristol Medical School, University of Bristol, UK
22. NIHR Bristol Biomedical Research Centre, UK
23. Health Data Research UK South-West, Bristol, UK
24. Department of Public Health and Epidemiology, RCSI University of Medicine and Health Sciences, Dublin, Ireland
25. Department of Mental Health, Johns Hopkins Bloomberg School of Public Health, Baltimore, MD, USA
26. University of Exeter Medical School, Exeter, UK

Corresponding Author

Harrison J Hansford

E: h.hansford@unsw.edu.au

School of Health Sciences, UNSW Sydney,

2052, Sydney, Australia

Words: 2247

For peer review only

Abstract

Background

Observational studies are increasingly used to inform health decision-making when randomised trials are not feasible, ethical, or timely. The target trial approach provides a framework to help minimise common biases in observational studies that aim to estimate the causal effect of interventions. Incomplete reporting of studies using the target trial framework limits the ability for clinicians, researchers, patients, and other decision-makers to appraise, synthesise, and interpret findings to inform clinical and public health practice and policy. This paper describes the methods that we will use to develop the transparent reporting of observational studies emulating a target trial (TARGET) reporting guideline.

Methods/design

The TARGET reporting guideline will be developed in five stages following recommended guidance. The first stage will identify target trial reporting practices by systematically reviewing published studies that explicitly emulated a target trial. The second stage will identify and refine items to be considered for inclusion in the TARGET guideline by consulting content experts using sequential online surveys. The third stage will prioritise and consolidate key items to be included in the TARGET guideline at an in-person consensus meeting of TARGET investigators. The fourth stage will produce and pilot-test both the TARGET guideline and explanation and

For peer review only

For peer review only - http://bmjopen.bmj.com/site/about/guidelines.xhtml

elaboration document with relevant stakeholders. The fifth stage will disseminate the TARGET guideline and resources via journals, conferences, and courses.

Ethics and Dissemination

Ethical approval for the survey has been attained (HC220536). The TARGET guideline will be disseminated widely in partnership with stakeholders to maximise adoption and improve reporting of these studies.

Key words: target trial emulation, causal inference, reporting guideline, observational studies

Strengths and Limitations

- The TARGET reporting guideline will be developed according to recommendations for health research reporting guidelines
- The TARGET working group has been established to include stakeholders from a variety of backgrounds
- A comprehensive piloting phase may increase the usability and uptake of the reporting guideline

For peer review only

Introduction

Observational studies can provide evidence on the causal effects of interventions when it is not feasible, ethical, or timely to conduct a relevant randomised trial. However, making causal inferences from observational data is challenging due to confounding and design-related biases such as selection bias and immortal time bias. (1,2) Design-related biases can be avoided using the target trial framework. (3,4) The framework involves the specification of the hypothetical randomised pragmatic trial — the target trial — that would ideally be conducted and how this trial might be emulated using observational data. (3,4) The two stages of the target trial framework are 1) specification of the target trial, and 2) emulation of the target trial. (3,4) Using observational data to mimic a randomised experiment was proposed in the mid 20th century, (5-8) and extended to time-varying treatments by Robins in 1986. (9)

The value of using the target trial framework to design the analysis of observational studies has been recognised by international regulatory bodies in the field of medicine and health, (10-14) and the framework underpins the widely-used ROBINS-I tool for assessing risk of bias in non-randomised studies of interventions. (15) Studies that are explicit in using the target trial framework have been published with increasing frequency in leading general medical and specialty journals. (16-23)

Application of the target trial framework requires the complete specification of the target trial protocol and its emulation (Figure 1). (3) Hernán & Robins (3) provide a

For peer review only - http://bmjopen.bmj.com/site/about/guidelines.xhtml

template for specifying a target trial and its emulation; however, there is currently no detailed guidance on reporting a study designed to emulate a target trial. Incomplete reporting of these studies limits the ability of clinicians, researchers, patients, and other decision-makers to appraise and synthesise findings or interpret them to inform clinical and public health practice and policy. A reporting guideline that expands upon the initial target trial emulation template(3) is needed to provide authors with comprehensive recommendations on how to completely and transparently report a study emulating a target trial.

For peer review only

[INSERT FIGURE 1]

To address this gap, we outline the processes and methods that used to develop a reporting guideline for studies emulating a target trial – TARGET (Transparent reporting of observational studies emulating a target trial).

Objective

The objective of the TARGET guideline is to provide guidance on the minimum set of items that should be reported to provide a clear and transparent account of observational studies that investigate the comparative effectiveness and safety of health interventions explicitly using the target trial framework.

Methods

We will develop the TARGET Guideline in five stages following recommendations for the development of health research reporting guidelines (Figure 2). (24) The start date for the study was late 2022, with the planned end date early 2025.

[INSERT FIGURE 2]

TARGET working group

The TARGET working group is made up of the steering committee and project team (Supplementary Material 1). The group was established to collate expertise on target trial emulation methodology, epidemiology, clinical trials, biostatistics, reporting guideline development, and knowledge of regulatory and journal editorial processes. The working group will oversee recruitment of participants for Stages 2 and 3 and contribute to writing and disseminating the guideline documents.

Stage 1: Identify current reporting practices

The systematic review aims to assess whether and how important items are reported by published studies explicitly emulating a target trial and whether reporting guidance (e.g., STROBE(25)) was used. The protocol for this systematic review was registered on the Open Science Framework on 13 March 2022 (osf.io/uj56m).

Databases, eligibility, and search terms

For peer review only

We will search Medline, EMBASE, PsycINFO and Science Citation Index for observational studies that stated in their methods that they explicitly emulated a target trial. We will exclude studies not written in English, not in the field of medicine and health, not conducted in humans, or not observational designs. Many observational studies may implicitly use the framework of a randomised trial. However, to be included in this review studies must be explicit in their attempt to emulate a target trial (e.g., stated 'target trial emulation' in the article). To identify eligible studies, we developed a literature search in collaboration with an expert librarian at the University of Oxford. Our approach used sensitive search terms including emulat*, target trial, observational data, real-world data, comparative effectiveness, and causal inference, to try to capture all papers explicitly emulating a target trial. The complete search strategy is in Supplementary Material 2. We will conduct forward citation tracking of selected seminal articles to maximise the chance of retrieving all relevant articles. (3,9,26-28) We will also include papers known to the authorship team. In duplicate, independent reviewers will conduct title, abstract, and full text screening. We will resolve disagreements between reviewers through discussion.

For peer review only

Data Extraction

We will extract items regarded by the steering committee as potentially important for the reporting of a target trial emulation, including those outlined by Hernán and Robins, 2016. (3) Two independent reviewers will extract information on study authors, year of publication, journal, sub-field of medicine, study design, sample size, intervention,

For peer review only - http://bmjopen.bmj.com/site/about/guidelines.xhtml

comparison group, outcomes assessed, and whether the study was prospectively registered. We will extract items relevant to the methods and results of the target trial emulation, including whether and how all components of the protocol of the proposed target trial, and how they were emulated, were specified (i.e., eligibility criteria, treatment strategies, assignment procedures, follow-up period, outcome(s), causal contrast(s), and data analysis plan). We will enter data into a standardised data extraction form which two authors will pilot with a selection of included studies. We will resolve disagreements in data extraction between reviewers through discussion, or where necessary, consultation with a third reviewer.

Data analysis

We will use R (29) for all data analyses. Categorical variables will be summarised using frequencies and percentages. Continuous variables will be summarised using mean and standard deviation, or median and interquartile range, as appropriate.

Outcomes of the systematic review

The systematic review will provide evidence on reporting in studies explicitly emulating a target trial. We acknowledge that excluding studies not written in English and unpublished studies may cause potentially relevant articles to be excluded. The findings will inform the online surveys (Stage 2) and the consensus meeting (Stage 3). We will submit the findings of this review for publication and all data and code made publicly available.

For peer review only - http://bmjopen.bmj.com/site/about/guidelines.xhtml

For peer review only

Stage 2: Identify and refine items for the TARGET guideline

We will conduct two online surveys to generate a list of candidate items that add detail to each of the protocol elements in Figure 1.

Ethics

Ethical approval has been obtained for the online surveys from the University of New South Wales Human Research Ethics Committee (HC220536).

Selection of initial items

The steering group will develop a list of key items, informed by the systematic review (Stage 1), and the target trial framework described by Hernán & Robins, (3) thought important for the conduct and reporting target trial emulations (Figure 1). Other potential sources of items include: published guidance for observational studies and randomised controlled trials, the ROBINS-I tool, (15) and studies that describe items that may be important for the conduct or reporting of target trial emulations.

Participants

Members of the TARGET working group (Supplementary Material 1) will be invited to participate in the surveys.

Procedure

For peer review only - http://bmjopen.bmj.com/site/about/guidelines.xhtml

We will host two online surveys using REDCap. (30,31) We will send each online survey via email to the participants. We will ask participants to rate the importance of each potential reporting item on a 9-point Likert scale (1, “not important”, to 9, “critically important”). Participants will have the opportunity to provide suggestions or modifications to the wording of items as well as suggest additional items or make other comments.

In the second survey, we will send participants a summary of the results for each potential reporting item (mean scores and standard deviations, median scores and interquartile ranges, and histograms), their own score for each item, and any comments from participants on each item from the first survey. We will also present any new items and suggested modifications to items. We will then invite participants to re-score the importance of each item, and score any additional items, considering the aggregated ratings. Participants will have the opportunity to provide additional feedback on each item in the form of open ended responses.

Analysis

Continuous variables will be summarised using mean and standard deviation, or median and interquartile range, as appropriate. We will analyse the free-text responses from the first and second surveys using an inductive approach, (32) in which we will use reflexive thematic (32) analysis to identify, organise and generate codes, and then identify themes found within the dataset. Briefly, inductive coding is a

For peer review only - http://bmjopen.bmj.com/site/about/guidelines.xhtml

process pooling common ideas without trying to fit ideas/codes into a pre-existing framework. These data will contribute to the creation of new items and modification of existing items to be included in the subsequent survey.

Outcome of the online surveys

We will generate a preliminary list of items with corresponding ratings of importance to be considered in the TARGET guideline at the consensus meeting (Stage 3). We will also generate qualitative insights to guide item refinement and prioritisation in preparation for the consensus meeting.

Stage 3 – Consolidate and prioritise key items to be included in the TARGET guideline

A consensus meeting will finalise reporting items for the TARGET guideline. (24) The consensus meeting will follow suggested methods for developing reporting guidelines, (24) including guidance for consensus-based methods currently being developed which we will use if they become available. (33)

Process

We will invite stakeholders identified by the working group to participate in a two-day consensus meeting. The TARGET working group will ensure that the expertise of consensus meeting participants includes target trial emulation methodology, epidemiology, clinical trials, biostatistics, reporting guideline development, and regulatory and journal editorial processes. Prior to the consensus meeting, the core

For peer review only

team will provide attendees with evidence from the systematic review (Stage 1) and findings from the online surveys (Stage 2) including a draft of the items proposed for inclusion in the guideline. We will present the findings from Stage 1 and 2 at the consensus meeting. A member of the TARGET working group will facilitate a structured discussion on the rationale for including items from the online surveys. If there are disagreements, they will first be debated and, if disagreements remain, we will hold an anonymised vote to establish the importance of including the item in the guideline. For the anonymised vote, a simple majority will be sufficient to guide the inclusion/exclusion of an item. The meeting will conclude with discussion about the content and production of relevant documents (TARGET guideline, draft explanation and elaboration document) as well as strategies for dissemination and implementation. Following the conclusion of the consensus meeting, we will circulate a report on the outcome to the meeting participants for review and approval.

Stage 4 – Development and piloting of the draft TARGET guideline and explanation and elaboration document

Stage 4 involves drafting the TARGET guideline and accompanying explanation and elaboration document to ensure that the wording and content of the documents are clear, precise, and suitable for all identified stakeholders. The purpose of the explanation and elaboration document is to explain each item by providing background information, a rationale, and clear reporting examples from published target trial emulations. We will design the explanation and elaboration document to facilitate

For peer review only

For peer review only - http://bmjopen.bmj.com/site/about/guidelines.xhtml

adherence to the TARGET guideline by clarifying the importance of each item, highlighting relevant reporting issues and providing examples to assist authors using the guideline. The consensus meeting participants may be asked to review and comment on the draft TARGET guideline and explanation and elaboration document.

We will evaluate the TARGET guideline by piloting the proposed guideline and the explanation and elaboration document with 20-30 expert methodologists and potential users of TARGET, identified from TARGET working group networks. We will ask participants to provide general feedback on accessibility and usability, and to identify possible reporting items that might have been overlooked. We will also ask for specific feedback about the utility and clarity of each TARGET item. We will collect data through online surveys, hosted by REDCap. (30,31) We will incorporate feedback from the piloting exercise into the final guideline and explanation and elaboration document, as required. If suggested revisions are extensive, we will conduct a further round of piloting.

Patient and public involvement

Potential users of this research include health researchers conducting observational analyses, regulatory bodies, public health and other health decision-makers. We aim to include relevant decision-makers in the piloting phase of the guideline development process to maximise the usefulness and uptake of the TARGET guideline. Participants

For peer review only

in any stage of the guideline development will be informed of the results and final guidance.

Stage 5 – Guideline implementation

The goal of the final stage of guideline development is to maximise reach and use of the TARGET guideline. The TARGET working group will guide the dissemination strategy with advice from consensus meeting participants. We aim to publish the TARGET guideline and the explanation and elaboration document and disseminate the findings through traditional and social media. We will engage journal editors and funding agencies to encourage TARGET guideline endorsement alongside other published reporting guidance. We will publicly host the TARGET guideline and explanation and elaboration paper, and any other relevant material on a TARGET website. We will index the guideline on the Enhancing the QUAlity and Transparency Of health Research (EQUATOR) Network website. (34,35) We will create online resources including infographics, blog posts and podcasts, which will be available on the TARGET website. We will share the TARGET guideline with authors in the field, and at relevant scientific conferences and methodological courses.

For peer review only

Declarations

Ethics approval and consent to participate

Not Applicable

Consent for publication

All authors consent to publication of this manuscript

Availability of data and materials

Not applicable

Funding

There was no specific funding for this study. HJH was supported by an Australian National Health and Medical Research Council (NHMRC) Postgraduate Scholarship, a PhD Top-Up Scholarship from Neuroscience Research Australia, and was a Neuroscience Research Australia PhD Pearl sponsored by Paul Salteri AO. AGC was supported by an Australian NHMRC Investigator Grant (ID 2010088). MM-B was supported by an Australian NHMRC Investigator Grant (ID 2009572). JMH was supported by an Australian NHMRC Investigator Grant (ID 2010128). BAD was supported by a grant from the National Institutes of Health (R00 CA248335). ME was supported by grants from the National Institutes of Health (R01 AI152772-01, 5U01-AI069924-05) and the Swiss National Science Foundation (32FP30-174281). NI was

For peer review only

For peer review only - http://bmjopen.bmj.com/site/about/guidelines.xhtml

supported by grants from the National Institute for Health and Care Research (HDRUK2022.0313) and the UK Office for National Statistics (2002563). MAH was supported by NIH grant R37 AI102634.

Competing interests

All authors declare no competing interests.

Author Contributions

HJH, AGC, MDJ, HL, JHM, conceived the idea for the project protocol. All authors contributed to the design and methodology of the project protocol. HJH and AGC wrote the first draft of the manuscript. MAH, SAS, IJD, BAD, XG-A, ME, RMG, NI, SL, MM-B, SAP, SS, JACS, MKS, EAS provided feedback, revised the manuscript and have read and approved the final version.

Acknowledgements

We acknowledge Nia Roberts, outreach librarian and information specialist at the University of Oxford for assistance designing the literature search.

For peer review only

Abbreviations

EQUATOR: Enhancing the QUAlity and Transparency Of health Research

REDCap: Research Electronic Data Capture

STROBE: Strengthening the Reporting of Observational Studies in Epidemiology

TARGET: TrAnsparent ReportinG of studies Emulating a Target trial

Figure Captions

Figure 1: Elements relevant to both the specification and emulation of the target trial described by Hernán & Robins (3)

Figure 2: Workflow for the development of the TARGET guideline

For peer review only

For peer review only - http://bmjopen.bmj.com/site/about/guidelines.xhtml

References

1. Hernán MA, Sauer BC, Hernández-Díaz S, et al. Specifying a target trial prevents immortal time bias and other self-inflicted injuries in observational analyses. *J Clin Epidemiol* 2016;79:70-75. doi: 10.1016/j.jclinepi.2016.04.014 [published Online First: 2016/05/31]
2. Dickerman BA, García-Albéniz X, Logan RW, et al. Avoidable flaws in observational analyses: an application to statins and cancer. *Nature Medicine* 2019;25(10):1601-06. doi: 10.1038/s41591-019-0597-x
3. Hernán MA, Robins JM. Using big data to emulate a target trial when a randomized trial is not available. *American journal of epidemiology* 2016;183(8):758-64.
4. Hernán MA. Methods of public health research—strengthening causal inference from observational data. *New England Journal of Medicine* 2021;385(15):1345-48.
5. Cochran WG. Observational studies. *Statistical papers in honor of George W Snedecor* 1972:77-90.
6. Dorn HF. Philosophy of inferences from retrospective studies. *American Journal of Public Health and the Nations Health* 1953;43(6_Pt_1):677-83.
7. Rubin DB. Estimating causal effects of treatments in randomized and nonrandomized studies. *Journal of Educational Psychology* 1974;66(5):688.
8. Wold H. Causality and econometrics. *Econometrica: Journal of the Econometric Society* 1954:162-77.
9. Robins J. A new approach to causal inference in mortality studies with a sustained exposure period—application to control of the healthy worker survivor effect. *Mathematical Modelling* 1986;7(9-12):1393-512.
10. Concato J, Stein P, Dal Pan GJ, et al. Randomized, observational, interventional, and real-world—What's in a name? *Pharmacoepidemiology and Drug Safety* 2020;29(11):1514-17.
11. Agency EM. European medicines agencies network strategy to 2025. The Netherlands: Health of Medicines Agencies, 2020.
12. Excellence NIfHaC. The NICE strategy 2021 to 2026, 2021.
13. Health Canada. Optimizing the Use of Real World Evidence to Inform Regulatory Decision-Making: Government of Canada, 2019.
14. Therapeutic Goods Administration. Real world evidence and patient reported outcomes in the regulatory context. https://www.tga.gov.au/review-real-world-evidence-and-patient-reported-outcomes: Australian Government, Department of Health, 2021.
15. Sterne JA, Hernán MA, Reeves BC, et al. ROBINS-I: a tool for assessing risk of bias in non-randomised studies of interventions. *BMJ* 2016;355:i4919. doi: 10.1136/bmj.i4919
16. Garcia-Albeniz XH, J.: Bretthauer, M.: Hernan, M. A. Effectiveness of screening colonoscopy to prevent colorectal cancer among medicare beneficiaries aged 70 to 79 years: A prospective observational study. *Annals of Internal Medicine* 2017 doi: http://dx.doi.org/10.7326/M16-0758
17. Mahévas M, Tran V-T, Roumier M, et al. Clinical efficacy of hydroxychloroquine in patients with covid-19 pneumonia who require oxygen: observational comparative study using routine care data. *BMJ* 2020;369:m1844. doi: 10.1136/bmj.m1844
18. Kaura A, Sterne JAC, Trickey A, et al. Invasive versus non-invasive management of older patients with non-ST elevation myocardial infarction (SENIOR-NSTEMI): a cohort

For peer review only - http://bmjopen.bmj.com/site/about/guidelines.xhtml

study based on routine clinical data. *The Lancet* 2020;396(10251):623-34. doi: 10.1016/S0140-6736(20)30930-2

19. Emilsson L, García-Albéniz X, Logan RW, et al. Examining Bias in Studies of Statin Treatment and Survival in Patients With Cancer. *JAMA Oncol* 2018;4(1):63-70. doi: 10.1001/jamaoncol.2017.2752
20. Chan You S, Krumholz HM, Suchard MA, et al. Comprehensive Comparative Effectiveness and Safety of First-Line β-Blocker Monotherapy in Hypertensive Patients: A Large-Scale Multicenter Observational Study. *Hypertension* 2021;77(5):1528-38. doi: 10.1161/hypertensionaha.120.16402 [published Online First: 20210329]
21. Caniglia EC, Robins JM, Cain LE, et al. Emulating a trial of joint dynamic strategies: An application to monitoring and treatment of HIV-positive individuals. *Stat Med* 2019;38(13):2428-46. doi: 10.1002/sim.8120 [published Online First: 20190318]
22. Zuo H, Yu L, Campbell SM, et al. The implementation of target trial emulation for causal inference: a scoping review. *J Clin Epidemiol* 2023 doi: 10.1016/j.jclinepi.2023.08.003 [published Online First: 20230808]
23. Scola G, Chis Ster A, Bean D, et al. Implementation of the trial emulation approach in medical research: a scoping review. *BMC Medical Research Methodology* 2023;23(1):186. doi: 10.1186/s12874-023-02000-9
24. Moher D, Schulz KF, Simera I, et al. Guidance for developers of health research reporting guidelines. *PLoS medicine* 2010;7(2):e1000217.
25. Von Elm E, Altman DG, Egger M, et al. The Strengthening the Reporting of Observational Studies in Epidemiology (STROBE) statement: guidelines for reporting observational studies. *Bulletin of the World Health Organization* 2007;85:867-72.
26. Hernán MA, Sauer BC, Hernández-Díaz S, et al. Specifying a target trial prevents immortal time bias and other self-inflicted injuries in observational analyses. *Journal of Clinical Epidemiology* 2016;79:70-75. doi: 10.1016/j.jclinepi.2016.04.014 [published Online First: 2016/05/31]
27. Hernán MA. How to estimate the effect of treatment duration on survival outcomes using observational data. *Bmj* 2018;360:k182. doi: 10.1136/bmj.k182 [published Online First: 2018/02/09]
28. Robins JM, Hernan, M. J. Estimation of the causal effects of time-varying exposures. In: Fitzmaurice G, Davidian M, Verbeke G, et al., eds. Longitudinal Data Analysis: CRC press 2008.
29. R Core Team. R: A language and environment for statistical computing. 2013
30. Harris PA, Taylor R, Minor BL, et al. The REDCap consortium: Building an international community of software platform partners. *Journal of Biomedical Informatics* 2019;95:103208. doi: https://doi.org/10.1016/j.jbi.2019.103208
31. Harris PA, Taylor R, Thielke R, et al. Research electronic data capture (REDCap)—A metadata-driven methodology and workflow process for providing translational research informatics support. *Journal of Biomedical Informatics* 2009;42(2):377-81. doi: https://doi.org/10.1016/j.jbi.2008.08.010
32. Braun V, Clarke V. Using thematic analysis in psychology. *Qualitative Research in Psychology* 2006;3(2):77-101. doi: 10.1191/1478088706qp063oa
33. Gattrell WT, Hungin AP, Price A, et al. ACCORD guideline for reporting consensus-based methods in biomedical research and clinical practice: a study protocol. *Research Integrity and Peer Review* 2022;7(1):3. doi: 10.1186/s41073-022-00122-0

For peer review only

34. The EQUATOR Network. EQUATOR Network - Enhancing the QUAlity and Transparency Of health Research 2022 [Available from: https://www.equator-network.org/2022.

35. Simera I, Moher D, Hoey J, et al. A catalogue of reporting guidelines for health research. *European journal of clinical investigation* 2010;40(1):35-53.

For peer review only

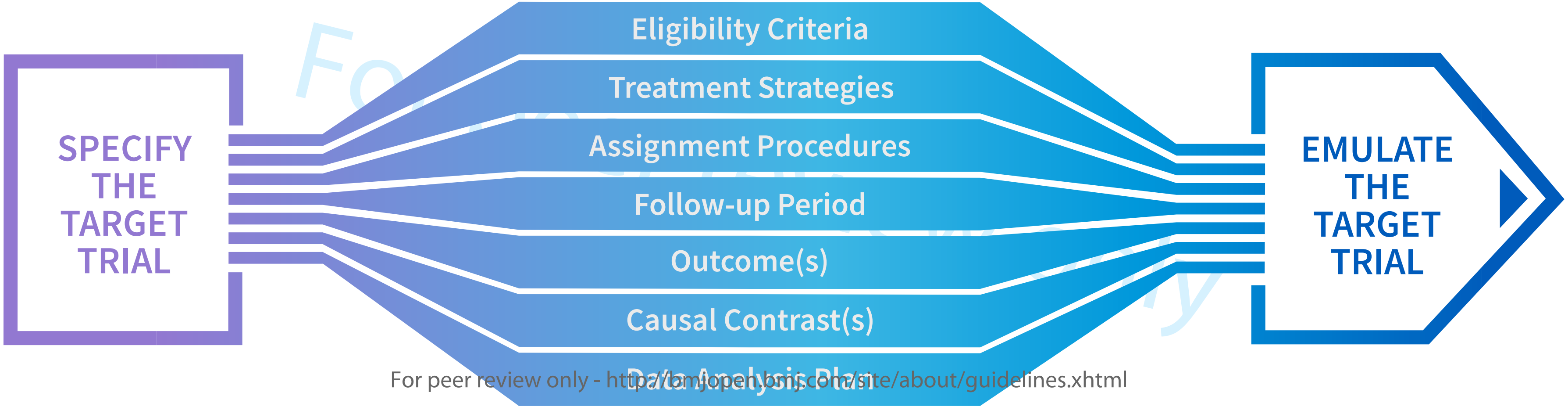

For peer review only - http://bmjopen.bmj.com/site/about/guidelines.xhtml

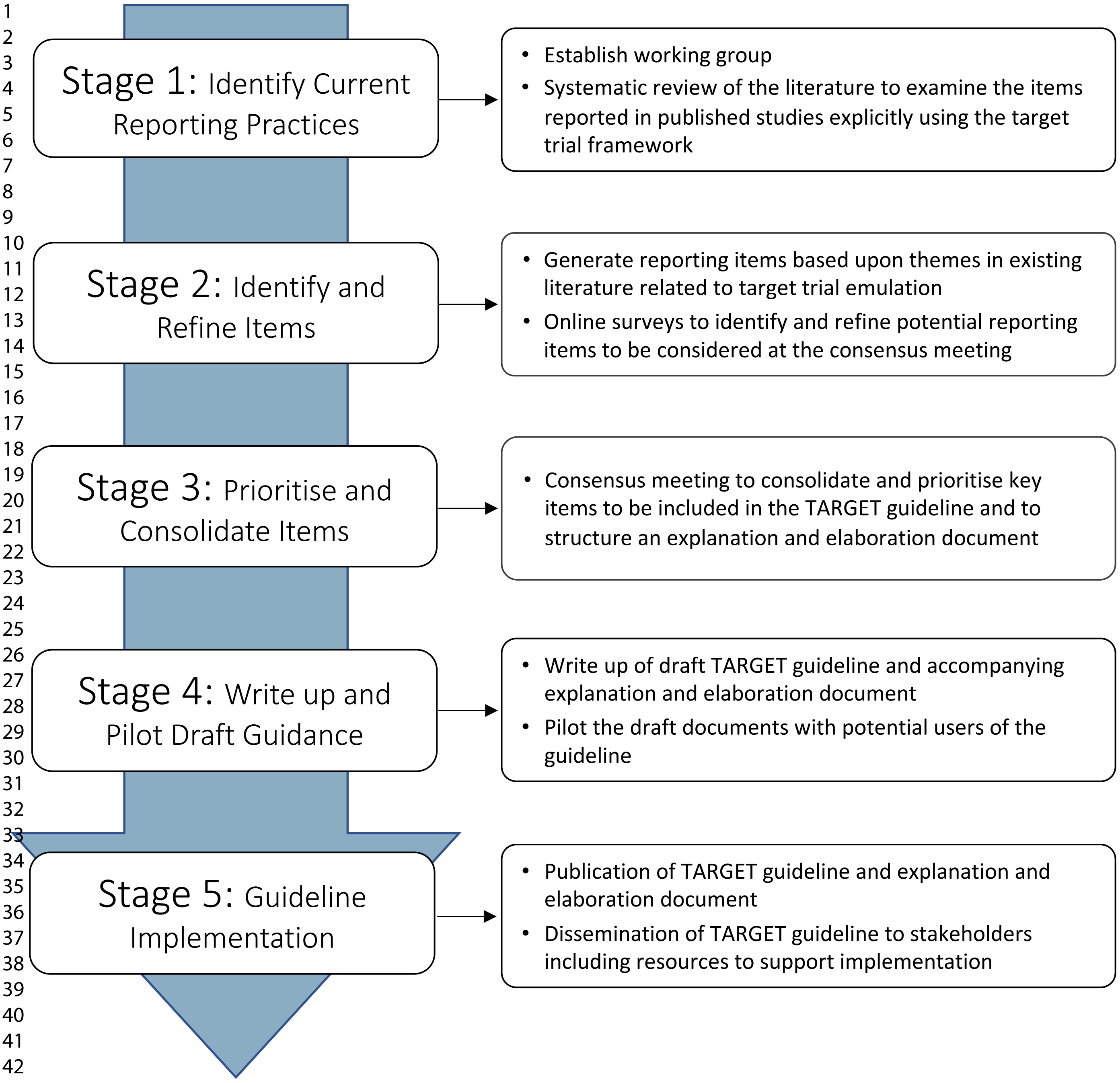
Stage 1: Identify Current Reporting Practices
Establish working group
Systematic review of the literature to examine the items reported in published studies explicitly using the target trial framework
Stage 2: Identify and Refine Items
Generate reporting items based upon themes in existing literature related to target trial emulation
Online surveys to identify and refine potential reporting items to be considered at the consensus meeting
Stage 3: Prioritise and Consolidate Items
Consensus meeting to consolidate and prioritise key items to be included in the TARGET guideline and to structure an explanation and elaboration document
Stage 4: Write up and Pilot Draft Guidance
Write up of draft TARGET guideline and accompanying explanation and elaboration document
Pilot the draft documents with potential users of the guideline
Stage 5: Guideline Implementation
Publication of TARGET guideline and explanation and elaboration document
Dissemination of TARGET guideline to stakeholders including resources to support implementation

Supplementary Material

Supplementary Material 1: TARGET working group members (alphabetical)

Steering committee
Dr Aidan G. Cashin
Mr Harrison J. Hansford
Prof Miguel A. Hernán
Dr Hopin Lee
Dr Matthew D. Jones
Prof James H. McAuley
A/Prof Sonja A. Swanson

Project team
A/Prof Issa J. Dahabreh
A/Prof Barbra A. Dickerman
Prof Matthias Egger
Dr Xabier Garcia-Albeniz
Prof Robert M. Golub
A/Prof Nazrul Islam
A/Prof Sara Lodi
A/Prof Margarita Moreno-Betancur
Prof Sallie A. Pearson
Prof Sebastian Schneeweiss
Prof Jonathan A. C. Sterne
Dr Melissa K. Sharp
Prof Elizabeth A. Stuart

For peer review only

Supplementary Material 2: Complete search strategies for all databases

Medline

1 (emulat* adj5 trial?).mp.
2 (target adj (trial? or experiment?)).mp.
3. (observational adj (stud* or research or data)).mp.
4. ((real world or rwd) adj2 (stud* or research or data)).mp.
5. (routine* adj2 data).mp.
6. (comparative effectiveness adj2 (stud* or research or data)).mp.
7. (emulat* or propensity score? or (causal adj2 (inference? or analys?s or effect*))).mp.
8. 3 or 4 or 5 or 6 or 7
9. 2 and 8
10. (target adj (trial? or experiment?)).ti.
11. 1 or 9 or 10
Filtered for time (2012-2022) manually after search

Embase

1. (emulat* adj5 trial?).mp.
2. (target adj (trial? or experiment?)).mp.
3. (observational adj (stud* or research or data)).mp.
4. ((real world or rwd) adj2 (stud* or research or data)).mp.
5. (routine* adj2 data).mp.
6. (comparative effectiveness adj2 (stud* or research or data)).mp.
7. (emulat* or propensity score? or (causal adj2 (inference? or analys?s or effect*))).mp.
8. 3 or 4 or 5 or 6 or 7
9. 2 and 8
10. (target adj (trial? or experiment?)).ti.
11. 1 or 9 or 10

psycINFO

noft(target trial emulat*) OR ((noft(real world data) OR (noft(emulat* trial)) OR noft(observational) OR noft(routine* data)) AND noft(comparative effective*) AND noft(causal infer*))

Web of Science

(TI=(emulat* trial)) OR (TI=(real world data) OR TI=(routine* data) OR TI=(comparative effectiveness study comparative effectiveness research or comparative effectiveness data) OR (TI=(emulat* or propensity score?) AND TI=(causal inference or causal analysis or causal effect*))) AND ALL=(target trial or emulat* or target trial emulation)

For peer review only
